# The pancancer overexpressed *NFYC* *Antisense 1* controls cell cycle mitotic progression through in *cis* and in *trans* modes of action

**DOI:** 10.1038/s41419-024-06576-y

**Published:** 2024-03-11

**Authors:** Cecilia Pandini, Giulia Pagani, Martina Tassinari, Emanuele Vitale, Eugenia Bezzecchi, Mona Kamal Saadeldin, Valentina Doldi, Giuliana Giannuzzi, Roberto Mantovani, Matteo Chiara, Alessia Ciarrocchi, Paolo Gandellini

**Affiliations:** 1 Department of Biosciences, University of Milan, Via Celoria 26, 20133 Milan, Italy; 2 Laboratory of Translational Research, Azienda USL-IRCCS di Reggio Emilia, Viale Risorgimento 80, 42123 Reggio Emilia, Italy; 3 Clinical and Experimental Medicine PhD Program, University of Modena and Reggio Emilia, Via Università 4, 41121 Modena, Italy; 4 Biology Department, School of Science and Engineering, The American University in Cairo, New Cairo, 11835 Egypt; 5 Molecular Pharmacology Unit, Department of Experimental Oncology, Fondazione IRCSS Istituto Nazionale dei Tumori, Via Amadeo 42, 20133 Milan, Italy; 6 Present Address: Department of Chemical and Biomolecular Engineering, University of Notre Dame, Notre Dame, IN 46556 USA

**Keywords:** Long non-coding RNAs, Non-small-cell lung cancer, Small-cell lung cancer

## Abstract

Antisense RNAs (asRNAs) represent an underappreciated yet crucial layer of gene expression regulation. Generally thought to modulate their sense genes in *cis* through sequence complementarity or their act of transcription, asRNAs can also regulate different molecular targets in *trans*, in the nucleus or in the cytoplasm. Here, we performed an in-depth molecular characterization of *NFYC* *Antisense* *1* (*NFYC-AS1*), the asRNA transcribed head-to-head to *NFYC* subunit of the proliferation-associated NF-Y transcription factor. Our results show that *NFYC-AS1* is a prevalently nuclear asRNA peaking early in the cell cycle. Comparative genomics suggests a narrow phylogenetic distribution, with a probable origin in the common ancestor of mammalian lineages. *NFYC-AS1* is overexpressed pancancer, preferentially in association with *RB1* mutations. Knockdown of *NFYC-AS1* by antisense oligonucleotides impairs cell growth in lung squamous cell carcinoma and small cell lung cancer cells, a phenotype recapitulated by CRISPR/Cas9-deletion of its transcription start site. Surprisingly, expression of the sense gene is affected only when endogenous transcription of *NFYC-AS1* is manipulated. This suggests that regulation of cell proliferation is at least in part independent of the in *cis* transcription-mediated effect on *NFYC* and is possibly exerted by RNA-dependent in *trans* effects converging on the regulation of G2/M cell cycle phase genes. Accordingly, *NFYC-AS1-*depleted cells are stuck in mitosis, indicating defects in mitotic progression. Overall, *NFYC-AS1* emerged as a cell cycle-regulating asRNA with dual action, holding therapeutic potential in different cancer types, including the very aggressive *RB1*-mutated tumors.

## Introduction

High-throughput sequencing has expanded our knowledge of the non-coding portion of human transcriptome, unearthing numerous novel transcripts [1, 2]. Among these, antisense RNAs (asRNAs), which originate from the antisense DNA strand and can overlap with the sense transcript of genes, are one of the most underappreciated classes [3]. Quantitative measurements of nascent transcripts support antisense transcription for up to ∼70% of protein-coding genes [4]. Despite the growing recognition for contributing to genome regulation, most asRNAs remain poorly characterized due to their very low abundance, which hinders accurate annotation [3]. From a functional perspective, asRNAs are thought to act in *cis* on their sense gene by transcription-dependent mechanisms and/or sequence complementarity [5–7]. Nevertheless, examples exist of asRNAs acting in *trans* and targeting genomic sequences distal to their locus in an RNA-dependent manner [3].

AsRNAs have been recognized as regulators of gene expression in multiple biological processes [7–10], and their aberrant expression/function is associated with tumorigenesis [11, 12]. AsRNAs may thus represent a rich and yet underexplored environment for the identification of cancer-relevant biomarkers and therapeutic targets. In this regard, the design of RNA-based therapies involving sequence-specific antisense oligonucleotide (ASOs) to target a pathogenic asRNA of interest (and in general long non-coding RNAs, lncRNAs) is quite straightforward [13]. In addition, as lncRNAs fold into tertiary structures forming domains with specific pockets, it is possible to target them using small molecules [14], which makes lncRNAs ideal entities to modulate processes linked to undruggable protein targets [14–16].

*NFYC* *Antisense 1* (*NFYC-AS1*, Supplementary Fig. S1A) is the asRNA transcribed head-to-head to *NFYC*, a subunit of the trimeric NF-Y transcription factor (TF). NF-Y has widely been described as a master regulator of proliferation in normal and tumor cells [17, 18]. In contrast, the information on *NFYC-AS1* is scanty. While it was shown upregulated in lung adenocarcinoma (LUAD), with its silencing able to induce cell death in LUAD cells via both apoptosis and autophagy [19, 20], crucial aspects of its biology remain to be elucidated. Here, we provide the first comprehensive characterization of *NFYC-AS1* expression pancancer, accompanied by a refined annotation of its transcript and an initial characterization of its cell cycle-regulated expression, role in supporting cell growth, and dual in *cis*/in *trans* action.

## Materials and Methods

### Cell culture

Established human tumor cell lines from American Type Culture Collection (ATCC, Rockville, MD, USA) were cultured in standard conditions, routinely tested for mycoplasma contamination using N-GRADE Mycoplasma PCR Reagent set (Euroclone S.p.A., Pero, Italy) and authenticated by STR profiling (Eurofins MWG-Biotech, Ebersberg, Germany). The NCI-H520 *RB1*-wildtype cell line (H520) is derived from a lung squamous carcinoma patient; these cells are adherent and have an epithelial morphology. The NCI-H82 *RB1*-mutated cell line (H82) is derived from a small cell lung carcinoma patient; H82 are epithelial cells that grow in suspension forming disordered aggregates. These cell lines were selected to model tumors where *NFYC-AS1* is up-regulated (including both *RB1* mutational backgrounds), since they show high levels of the asRNA (>4 TPM).

Both cell lines were grown in RPMI 1640 supplemented with 10% fetal bovine serum (FBS), 2 mM L-Glutamine and 25 mM Hepes (Euroclone S.p.A., Pero, Italy), at 37 °C in a 5% CO2 atmosphere. For H82 cells, the medium was also supplemented with 4500 mg/L D-Glucose (GeneSpin Srl, Milan, Italy) and 1 mM Sodium Pyruvate (NaPyr, Sigma-Aldrich, Saint Louis, MI, USA).

### 5’ and 3’ RACE

The 5’ and 3’ RACE System for Rapid Amplification of cDNA Ends Version 2.0 (Thermo Fisher Scientific Inc., Waltham, MA, USA) was used to characterize *NFYC-AS1* 5*’* and 3’ ends in H520 cells, according to manufacturer’s instructions. RACE products were sequenced-verified by Eurofins MWG-Biotech (Ebersberg, Germany).

### Gapmer ASO and siRNA treatment

Six different Gapmer ASOs (QIAGEN, Hilden, Germany), GAP1 to GAP6, were designed to target different portions of *NFYC-AS1* putative transcript (relative positions are shown in Supplementary Fig. S3A and sequences reported in Supplementary Table S9), making sure that they did not overlap any annotated repetitive regions. Gapmers were transfected at 5-25 nM concentration in Opti-MEM™ (Thermo Fisher Scientific Inc., Waltham, MA, USA) using Lipofectamine™2000 (Thermo Fisher Scientific Inc., Waltham, MA, USA). 7 ×10^5^ H520 cells were seeded the day before transfection, transfected for 6 h with Gapmers or the Negative Control Gapmer (NEG), then incubated in complete medium for 48/72 h. For H82 cells, the day of transfection, 1.0 ×10^6^ H82 cells were transfected in their medium and incubated for 48/72 h. The same protocols were applied to transfect 40 nM *NFYB* siRNA and relative control siRNA (siCT).

### *NFYC-AS1* Transcription Start Site (TSS) deletion by CRISPR/Cas9

Two sgRNAs targeting *NFYC-AS1* TSS region were designed and cloned into PLV-Cas9-T2A-GFP plasmid (Addgene #53190 - Cambridge, MA, USA). H520 cells were transfected with 2.5 µg plasmids using Lipofectamine™2000. GFP^+^ cells were sorted using BD FACSAria II flow cytometer and cultured as single clones. Genomic DNA from single clones was extracted using QuickExtract^TM^ DNA Extraction Solution (Lucigen by Biosearch Technologies, Hoddesdon, UK) and 2x Master Mix Standard GL kit (GeneSpin Srl, Milan, Italy) was used to perform end-point PCR, and PCR products verified for the deletion on agarose gel.

### Cell-cycle synchronization and re-entry

2 × 10^6^ H520 cells per time point were seeded in complete medium. The day after, cells were left in a serum-deprived medium (0% FBS) for 48 h. Synchronous cell-cycle re-entry was stimulated by adding complete medium (10% FBS). Cells were harvested at different time points after serum addition, and cyclin mRNA levels measured by qRT-PCR to define cell-cycle phases.

### RNA-sequencing

RNA-sequencing was performed in quadruplicates on H520 cells treated with GAP2/3/4 compared to NEG at 48 h after transfection and on four independent ΔTSS H520 clones compared to three independent Wild-Type (WT) clones. Polyadenylated RNA was purified through oligo-dT-based RNA capturing, randomly fragmented, and transformed into cDNA using random hexamers with NEB library preparation protocol. Library preparation, paired-end sequencing, and data quality control were performed by Novogene, UK. Gene expression levels were computed by RSEM-1.3.1 software using NCBI RefSeq (GRCh38/hg38) as a reference. Bioinformatic analyses are described in Supplementary Methods.

### Statistics and reproducibility

The results are presented as mean values ± standard deviation (sd) or standard error (se). *p*-values were calculated by unpaired two-tailed *t*-test, one sample *t*-test, Jonckheere-Terpstra test, or log-rank test as highlighted in captions (where the number of replicates for the different experiments is indicated) and considered statistically significant if *p* < 0.05.

### Additional Materials and Methods

Additional methods are described in Supplementary Methods. Sequences of primers, Gapmers and siRNAs, and sgRNAs are reported in Supplementary Tables S1-S3. Original data of qRT-PCR and western blot are reported as Original Data 1 and 2, respectively.

## Results

### *NFYC-AS1* is upregulated in cancer with preferential association with *RB1* mutation

**Fig. 1:**
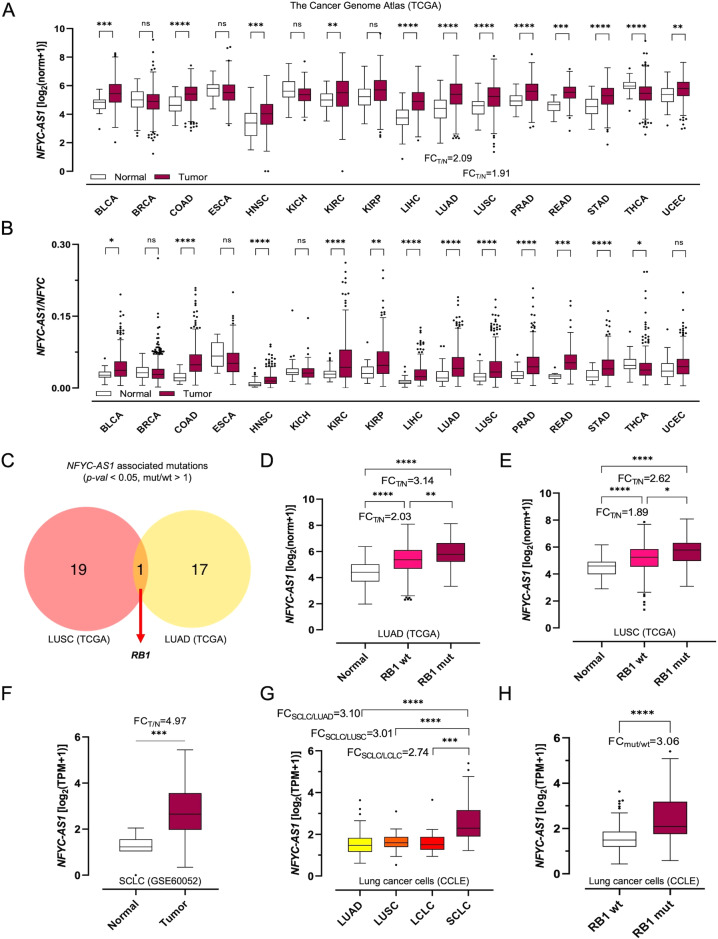
*NFYC-AS1* expression in tumor tissues and cells. **A** Boxplots of *NFYC-AS1* expression and **B** *NFYC-AS1*/*NFYC* expression level ratios in tumor and matched control tissues (TCGA). **C** Venn diagram showing the intersection between the top significant mutations (sorted by *p*-value) associated with higher *NFYC-AS1* expression level (mut/wt > 1) in LUAD and LUSC (TCGA). **D** Boxplots of *NFYC-AS1* expression level in *RB1*-wild-type (*RB1*-wt) and *RB1*-mutated (*RB1*-mut) tumors compared to normal tissues in LUAD and **E** LUSC (TCGA). **F** Boxplots of *NFYC-AS1* expression in SCLC tumors compared with normal tissues (GSE60052). **G** Boxplots of *NFYC-AS1* expression in lung cancer CCLE cell lines according to the lung cancer histotype of origin or **H** the *RB1* mutational status. Throughout the figure, the gene expression level is expressed as logarithm in base 2 of the normalized counts (norm) or TPM plus one [log_2_(norm or TPM + 1)], depending on the available data. The tumor-normal fold-change (FC_T/N_) is calculated as the ratio between the average normalized counts or TPM for *NFYC-AS1* in tumors and in normal tissues, in SCLC and in the other lung cancer histotypes, or in presence and absence of *RB1* mutation. The *NFYC-AS1*/*NFYC* ratio is calculated as the ratio between the normalized counts for *NFYC-AS1* and for *NFYC* in individual samples and then averaged. Two-tailed unpaired *t*-test *p*-values are reported, *p < 0.05, **p < 0.01, ***p < 0.001, ****p < 0.0001, ns (non-significant).

Across healthy human tissues, *NFYC-AS1* expression levels are high in cerebellum, uterus, prostate, thyroid, cervix, ovary (Supplementary Fig. S1B), overall mirroring those of its sense gene *NFYC* (Supplementary Fig. S1C), though remaining invariably lower (*NFYC-AS1*/*NFYC* ratio=0.0923-0.365). Analysis of The Cancer Genome Atlas (TCGA) [21] showed a systematic upregulation of *NFYC-AS1* in most human tumors as compared to normal tissues, including the main histotypes of non-small cell lung cancer (NSCLC), lung adenocarcinoma (LUAD) and lung squamous carcinoma (LUSC) (Fig. 1A). Conversely *NFYC* modulation is not reproducible across cancer types, with some tumors showing concomitant *NFYC-AS1* upregulation and *NFYC* downregulation (Supplementary Fig. S2A). Accordingly, *NFYC-AS1*/*NFYC* expression ratio (calculated in individual samples) is increased in most human cancers (Fig. 1B), suggesting a tumor-specific asRNA overexpression. Moreover, *NFYC-AS1* levels are sufficient to discriminate between normal and tumor samples in NSCLC cohorts and pancancer (Supplementary Fig. S2B). *NFYC-AS1* overexpression in NSCLC was confirmed in an independent dataset (GSE81089), with superimposable tumor/normal fold-changes (Supplementary Fig. S2C). No major differences were found in *NFYC-AS1* expression across NSCLC molecular subtypes, with the exception of markedly higher levels in LUSC primitive subtype (Supplementary Fig. S2D, E). Instead, a significant association was found with selected mutational profiles: lower asRNA levels were observed in *KRAS*-mutated (-mut) LUAD and in *NF1*/*MYCBP2*-mut LUSC (Supplementary Tables S4, 5). Mutation in *RB1*, a prototypical tumor suppressor gene [22], was found significantly associated with increased *NFYC-AS1* expression in both LUAD and LUSC (Fig. 1C–E), where it affects 5% and 7% of cases, respectively. Notably, 14% of primitive LUSC, the form with the worse prognosis [23], are *RB1*-mut (Supplementary Fig. S2E), consistent with the particularly high *NFYC-AS1* levels observed in this subtype.

*RB1* gene mutations are generally rare but reach 90% in the highly aggressive small cell lung cancer (SCLC) histotype [24], where they drive tumor cell hyperproliferation and increased lineage plasticity towards a neuroendocrine phenotype [25]. We indeed found that *NFYC-AS1* is markedly upregulated in SCLC (Fig. 1F), with a tumor-normal fold-change of 4.97, the highest among the lung cancer histotypes analyzed in this study. This finding was corroborated by the analysis of 208 lung cancer cell lines from the Cancer Cell Line Encyclopedia (CCLE) [26], where a major upregulation of *NFYC-AS1* is observable in SCLC compared to LUAD, LUSC and large cell lung carcinoma (LCLC) cells (Fig. 1G), and in *RB1*-mut vs -wt cells (Fig. 1H). Considering SCLC cell line classification into molecular subtypes [25], higher *NFYC-AS1* levels were found in neuroendocrine ASCL1 and NEUROD1 groups (Supplementary Fig. S2F), which are characterized by 71% and 90% of *RB1*-mut cell lines, respectively, as compared to the YAP1 subtype, which has low/absent expression of neuroendocrine markers [27] and comprises only 15% of *RB1*-mut cell lines. Moreover, *NFYC-AS1* levels were sufficient to discriminate between *RB1*-wt and *RB1*-mut NSCLC tumors and lung cancer cell lines, with good levels of accuracy (Supplementary Fig. S2G).

### Accurate transcript annotation reveals a dominant long *NFYC-AS1* isoform and prevalently nuclear expression

**Fig. 2:**
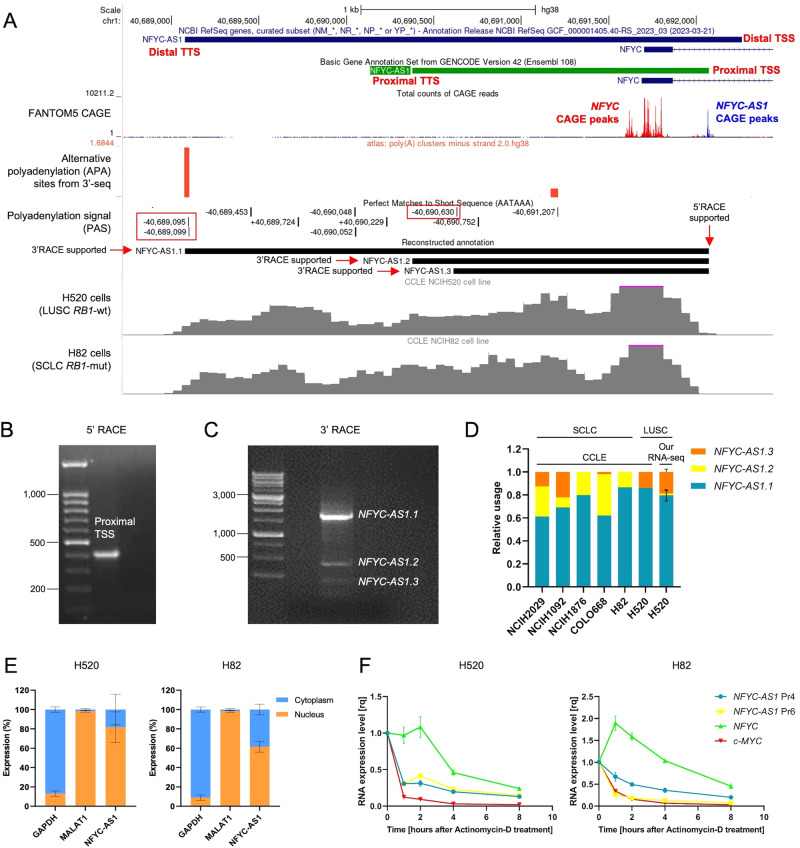
*NFYC-AS1* transcript reannotation and subcellular localization. **A** *NFYC(-AS1)* locus at chr1p34.2 as from UCSC Genome Browser (GRCh38/hg38 assembly). From the top to the bottom: NCBI RefSeq and GENCODE V42 annotations are reported together with CAGE peaks (FANTOM5 project) on the minus strand for *NFYC-AS1* (blue) and plus strand for *NFYC* (red), polyadenylation sites on the minus strand (red) (polyA site database, 3’-seq data), polyadenylation signal (PAS), our *NFYC-AS1* reconstructed annotation, and BigWig profile for H520 and H82 cell lines. **B** Electrophoretic gel of 5’RACE PCR products. **C** Electrophoretic gel of 3’RACE PCR products. **D** Bar plot showing the relative abundance of *NFYC-AS1* isoforms in different cell lines from the CCLE and in H520 cell line as from our RNA-seq. **E** Bar plot showing the relative percentage of *NFYC-AS1* measured through qRT-PCR using primer 4 (Pr4) in the nuclear and cytoplasmic fractions of H520 and H82 cells. *MALAT1* and *GAPDH* are used as control for the nuclear and cytoplasmic fractions, respectively. **F** Expression levels of *NFYC-AS1*, *NFYC* and *C*-*MYC* (qRT-PCR) in H520 and H82 cell lines, after Actinomycin-D treatment. Data are normalized to time 0 for every gene and represent the mean ± sd, as from *n* = 3 independent qRT-PCR measurements.

A monoexonic transcript is reported for *NFYC-AS1* both in the Refseq and Gencode annotations, but marked differences exist in the designation of the TSS, termination site (TTS), and RNA length (Supplementary Fig. S1A). By analyzing the profile of RNA-seq reads from cell lines with high levels (>4 TPM) of the asRNA (H520 LUSC cells and H82 SCLC cells), we observed a good support for the Gencode TSS (proximal TSS, w.r.t. the *NFYC* TSS) (Fig. 2A). Two FANTOM CAGE [28] peaks were found next to only the proximal TSS, which also corresponded to the peak of DNase I hypersensitivity signal (Fig. 2A). Furthermore, 5’RACE experiments indicated the proximal TSS as the true 5’ end (Fig. 2A, B, Supplementary Fig. S3A).

Three different polyadenylated 3’ ends were identified by 3’RACE and confirmed by Sanger sequencing (Fig. 2A, C, Supplementary Fig. S3A), corresponding to the RefSeq, the Gencode and an additional TTS, named *NFYC-AS1.1* (long), *NFYC-AS1.2* (medium) and *NFYC-AS1.3* (short). A canonical AAUAAA/AUUAAA polyadenylation signal (PAS) was indeed found upstream of the long and short 3’ ends (Fig. 2A), while 11 A’s were found at the intermediate TTS. To exclude mispriming in 3’RACE, oligo-dT and random hexamer retrotranscribed RNA were compared using different primers (Supplementary Fig. S3A): similar oligo-dT/random hexamer ratios were observed for primers in proximity of all the putative TTSs (Supplementary Fig. S3B), confirming them as true 3’ ends. Re-analyzing RNA-seq data from highly expressing cells using our refined annotation, long *NFYC-AS1* isoform resulted to be the most abundant overall, nevertheless low levels of expression of short and medium isoforms were observed in some samples (Fig. 2D), consistent with 3’-seq data from the PolyASite database [29] (Fig. 2A).

*NFYC-AS1* subcellular localization was assessed in H520 and H82 cells, revealing a prevalently nuclear expression (Fig. 2E). Although five complete ORFs of > 75 nt in size can be detected within *NFYC-AS1* sequence, computational prediction, and interrogation of publicly available RIBO-seq data consistently classified the transcript as devoid of protein-coding potential (Supplementary Fig. S3C and Supplementary Tables S6–10), ultimately indicating that *NFYC-AS1* is a lncRNA. Decay experiments showed that *NFYC-AS1* transcript half-life is less than one hour, similarly to the short-lived transcript *C*-*MYC* [30]; however, detectable levels were observed at later time points, suggesting that *NFYC-AS1* turnover is rapid, though a pool of stable transcripts may exist (Fig. 2F). In contrast, *NFYC* mRNA revealed to be quite stable with a lower decay (Fig. 2F).

### *NFYC-AS1* is regulated by NF-Y and conserved throughout mammals

**Fig. 3:**
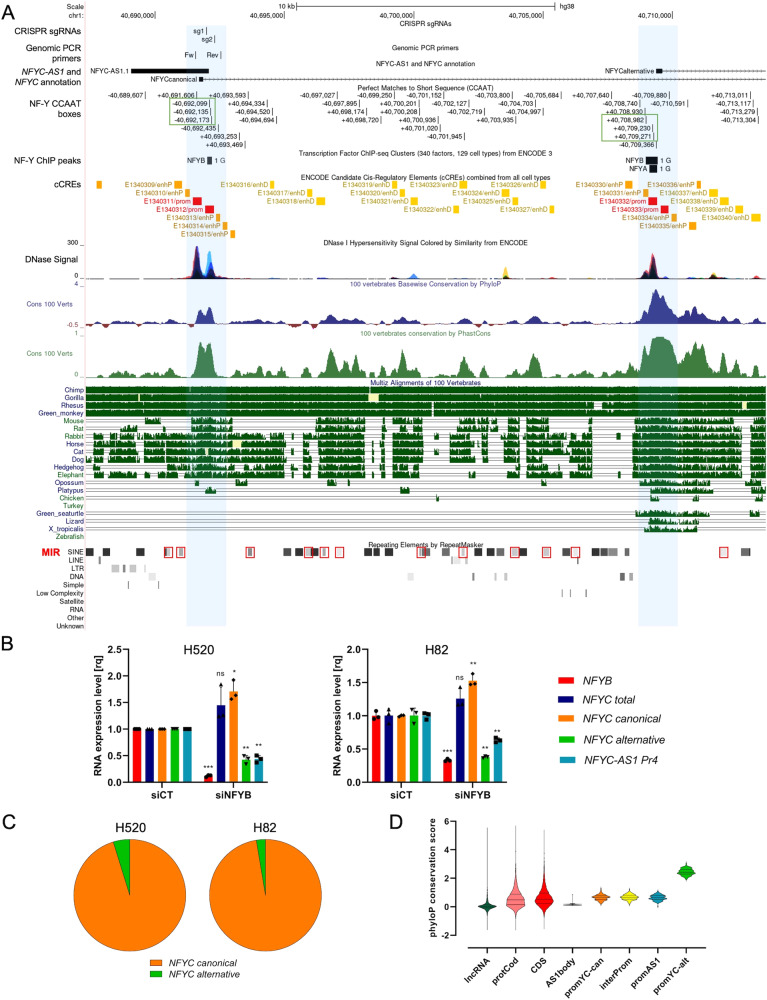
Analysis of *NFYC-AS1* conservation and regulation by NF-Y. **A** *NFYC(-AS1)* locus at chr1p34.2 as from UCSC Genome Browser (GRCh38/hg38 assembly). Top: CRISPR/Cas9 single guide (sg)RNAs and genomic PCR primers are shown together with the long *NFYC-AS1* isoform. Middle: transcription regulatory elements, including CCAAT boxes (green rectangles), NF-Y ChIP-seq peaks (ENCODE), ENCODE candidate *Cis*-Regulatory Elements (cCREs), and DNase hypersensitivity tracks. Bottom: a detailed overview of conservation profiles according to the Vertebrate Multiz Alignment & Conservation (100 Species) track in the Genome Browser. Blue: phyloP conservation score. Green: PhastCons conservation score. Alignment of a manual selection of representative genome sequences for different taxa is represented at the bottom, together with the Repeating Element by RepeatMasker track; mammalian-wide interspersed repeats (MIRs) are highlighted by red rectangles. **B** Bar plot showing *NFYB, NFYC* total, *NFYC* canonical, *NFYC* alternative, and *NFYC-AS1* expression levels (qRT-PCR) at 48 h after transfection with *NFYB* siRNA (siNFYB) in H520 *RB1*-wt cells and H82 *RB1*-mut cells. Data are siCT-normalized and reported as mean ± sd, as from *n* = 3 independent biological replicates. One sample *t*-test *p*-values are reported. *p < 0.05, **p < 0.01, ***p < 0.001, ****p < 0.0001, ns (non-significant). **C** Pie charts showing the cumulative fraction of *NFYC* transcripts originating from either TSS (*NFYC* canonical and alternative) as from RNA-seq data of H520 and H82 cells. **D** PhyloP conservation score at exons of lncRNAs (lncRNAs), exons of protein coding genes (protCod), protein coding exons of protein coding genes (CDS), compared with different *NFYC-AS1* segments/regions and *NFYC* regulatory elements: AS1body, *NFYC-AS1* 3’ end segment not overlapped with *NFYC*; promYC_can, canonical *NFYC* isoform promoter; interProm, *NFYC-AS1* segment spanning the genomic region in between *NFYC* canonical promoter and *NFYC-AS1* promoter; promAS1, *NFYC-AS1* promoter; promYC_alt, alternative *NFYC* isoform promoter. Promoters were defined as the genomic region spanning 250 bp upstream of annotated transcription start sites.

ENCODE DNase I hypersensitivity data were inspected to annotate *NFYC-AS1* and *NFYC* regulatory elements, revealing two peaks corresponding to *NFYC* TSS and *NFYC-AS1* proximal TSS (Fig. 3A). According to ENCODE candidate *cis*-regulatory elements (cCREs), two clearly distinct promoters insist on this genomic region, indicating that the two genes are transcribed independently (Fig. 3A). Three equidistant CCAAT boxes – a typical feature of promoters bound by NF-Y TF [17] – and NF-Y ChIP-seq signal were found just upstream of *NFYC-AS1* TSS (Fig. 3A); consistent with this, abrogation of NF-Y activity through *NFYB* silencing resulted in a dramatic downregulation of *NFYC-AS1* in both H520 and H82 cells (Fig. 3B). In contrast, none of these features is present immediately upstream of *NFYC* TSS (Fig. 3A), and, accordingly, *NFYC* was not repressed upon *NFYB* silencing (Fig. 3B). Although *NFYC* has an alternative TSS, approximately 17 kb downstream of *NFYC-AS1* (Fig. 3A), this TSS was shown to be induced only upon DNA damage [31] and is considerably less used in basal conditions compared with the canonical TSS, as exemplified by the cumulative fraction of *NFYC* transcripts originating from either TSS in H520 and H82 cells (Fig. 3C). Curiously, the alternative TSS resulted to be directly under the control of NF-Y, as *NFYB* silencing markedly abrogated the expression of alternative *NFYC* transcript in both cell lines (Fig. 3B), in line with the presence of CCAAT boxes and NF-Y ChIP-seq peaks in its proximity (Fig. 3A).

*NFYC-AS1* sequence is highly conserved in primates and moderately conserved in other mammals, with the exception of mouse/rodents (Fig. 3A). An orthologue asRNA with similar tissue expression pattern as in humans has been described in dog [32]. Inspection of conservation tracks and multiple alignments of genome sequences showed that *NFYC* alternative TSS has high levels of conservation across vertebrates; conversely, the canonical TSS is conserved only in mammals (Fig. 3A, D). *NFYC-AS1* TSS displayed levels of sequence conservation comparable to those of *NFYC* first exon and canonical TSS, with a score in line with that of protein-coding exons (Fig. 3D). Lower levels of conservation were instead observed for *NFYC-AS1* 3’ terminal portion, which were however comparable with those of other lncRNAs (Fig. 3D).

Interestingly, both *NFYC-AS1* and *NFYC* first intron are punctuated by Mammalian-wide Interspersed Repeats (MIRs) (Fig. 3A), a family of transposable elements specific to mammals that have been linked with the dissemination of novel promoters and enhancer elements throughout mammalian and human genomes [33]. This arrangement and the observed patterns of sequence conservation might indicate that *NFYC* canonical TSS/first intron and *NFYC-AS1* originated in mammals, while the broader phylogenetic distribution and higher levels of conservation would position *NFYC* alternative TSS as ancestral.

### *NFYC-AS1* knockdown impairs proliferation of both *RB1*-wt and -mut cancer cells

**Fig. 4:**
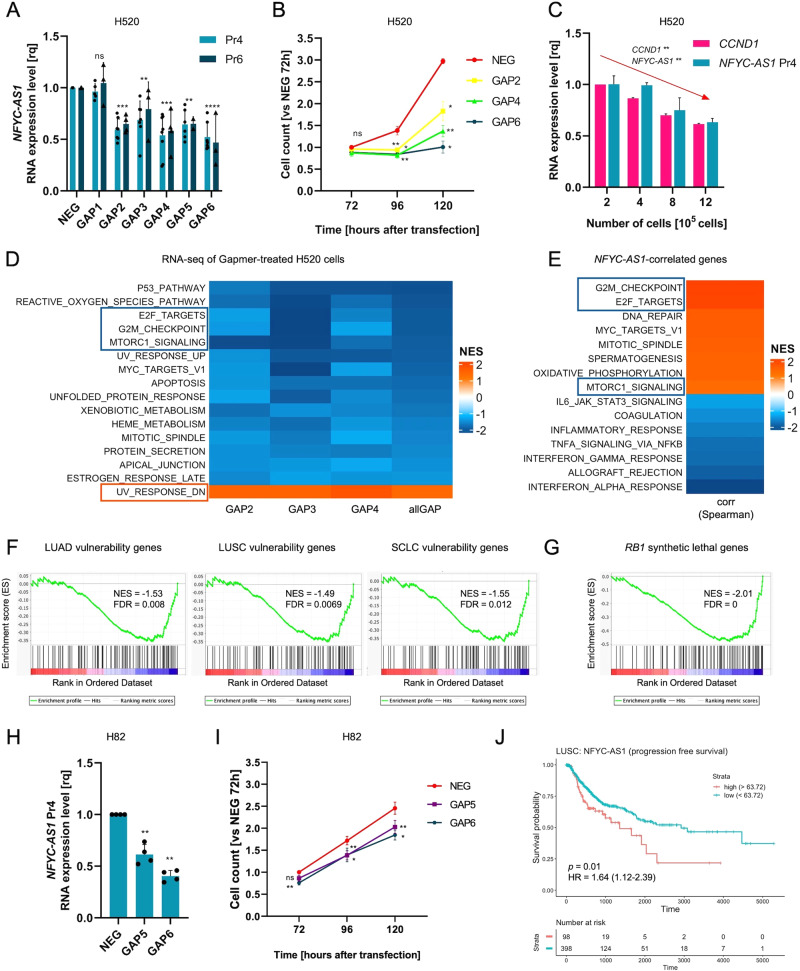
Characterization of *NFYC-AS1* knockdown phenotype by Gapmer ASOs. **A** Bar plot showing *NFYC-AS1* expression measured through qRT-PCR using primer 4 (Pr4) and primer 6 (Pr6) at 48 h after 5 nM Gapmer transfection in H520 *RB1-*wt cells. Data are NEG-normalized and reported as mean ± sd as from multiple independent biological replicates (*n* indicated in Supplementary Table S11). **B** Cell counts at different timepoints after *NFYC-AS1* Gapmer-knockdown, normalized against the NEG at 72 h in H520 cells. Data reported as mean ± se, as from *n* = 3 independent biological replicates. **C** Bar plot showing *NFYC-AS1* and *CCND1* expression levels (qRT-PCR) in H520 cells seeded at different cell densities, normalized against the lowest confluency. Data reported as mean ± sd, as from *n* = 3 independent biological replicates. Jonckheere-Terpstra test *p*-values are shown. **D** Heatmap reporting NES (FDR < 0.10 for all three Gapmers) of cancer hallmarks (GSEA) for genes differentially expressed in Gapmer-transfected H520 cells (allGap = all Gapmers vs NEG). **E** Heatmap reporting NES (FDR < 0.10) of cancer hallmarks (GSEA) for genes ranked for correlation with *NFYC-AS1* in LUSC cells (CCLE). **F** GSEA plots of genes related to lung cancer vulnerabilities [34] or **G** synthetic lethal in *RB1*-mut cells [35] in genes modulated upon *NFYC-AS1* Gapmer-silencing. **H** Bar plot showing *NFYC-AS1* expression (qRT-PCR) at 48 h after 25 nM Gapmer transfection in H82 *RB1*-mut cells. Data are NEG-normalized and reported as mean ± sd, as from *n* = 4 independent biological replicates. **I** Cell counts at different time points after *NFYC-AS1* Gapmer-knockdown, normalized against NEG at 72 h in H82 cells. Data reported as mean ± se, as from *n* = 6 independent biological replicates. **J** Progression-free survival curve stratified according to Cutoff Finder-determined threshold (63.72) for *NFYC-AS1* in LUSC patients (TCGA). Log-rank test *p*-value and hazard risk (HR) are shown. *p < 0.05, **p < 0.01, ***p < 0.001, ****p < 0.0001, ns (non-significant). One sample *t*-test *p*-values are reported for panels **A**, **B**, **H**, and **I**.

Six different Gapmer ASOs were used to knockdown *NFYC-AS1* in *RB1*-wt H520 cells, three of which (GAP2, GAP3, and GAP4) targeted all *NFYC-AS1* isoforms, two (GAP5 and GAP6) only the long isoform, and one (GAP1) the sequence in between the proximal and distal TSS (Supplementary Fig. S3A). Gapmers from 2 to 6 effectively downmodulated *NFYC-AS1* (Fig. 4A), with a degree of repression that paralleled transcript accessibility (Supplementary Fig. S4A, B). GAP1, despite the high accessibility of its target region, failed to repress *NFYC-AS1*, again supporting the use of the proximal TSS (Fig. 4A, Supplementary Fig. S4A). *NFYC-AS1* silencing resulted in a time-dependent reduction of cell growth, which was proportional to the extent of knockdown exerted by the different Gapmers (Fig. 4B). As a control, GAP1 did not induce any significant decline of cell number (Supplementary Fig. S4C). The other way around, we found that *NFYC-AS1* levels decreased endogenously in H520 cells as they reduced their growth in response to confluency, in trend with cyclin D1 levels (Fig. 4C). Altogether, these results suggest a role of *NFYC-AS1* in supporting cell proliferation.

To frame transcriptome changes induced by *NFYC-AS1* silencing, RNA-seq was performed on cells treated with GAP2, GAP3, and GAP4. GSEA analysis performed on ranked gene lists from individual Gapmers and all Gapmers together (compared to NEG) revealed significant enrichment of proliferation/cell cycle-related gene sets, such as *E2F targets*, *G2/M checkpoint* and *mTORC1 signaling* in downregulated genes (Fig. 4D). Following a guilt-by-association approach, the same gene sets were markedly enriched in genes positively correlated with *NFYC-AS1* in LUSC cells (Fig. 4E), suggesting a physiological link between the asRNA and proliferation-related programs.

Interestingly, genes downregulated upon *NFYC-AS1* knockdown were enriched in possible actionable therapeutic targets for all lung cancer histotypes, as identified from DepMap vulnerabilities [34] (Fig. 4F). A significant enrichment in genes that are synthetic lethal in *RB1*-mut SCLC cells was also observed (signatures from *Oser* et al. [35].) (Fig. 4G), suggesting that *NFYC-AS1* inhibition may have therapeutic effects in SCLC.

To test this hypothesis, we used Gapmers to silence *NFYC-AS1* in *RB1*-mut H82 cells. The in-suspension growth pattern and the poor transfectability of these cells allowed only very modest knockdown with 5 nM Gapmers (Supplementary Fig. S4D), whereas 25 nM of the most effective Gapmers, GAP5 and GAP6, recapitulated *NFYC-AS1* repression obtained in H520 cells (Fig. 4H). In this setting, both Gapmers significantly reduced H82 cell growth at all time points, even if at a lesser extent as compared to H520 cells (Fig. 4I).

In accord with the observed growth-supporting function of *NFYC-AS1*, survival analyses revealed that its higher expression in tumors is associated with significantly increased risk of disease progression in LUSC patients (Fig. 4J) and a trend for decreased overall survival in SCLC patients (Supplementary Fig. S4E).

### *NFYC-AS1* genetic editing recapitulates growth impairment phenotype revealing a role in mitotic progression

**Fig. 5:**
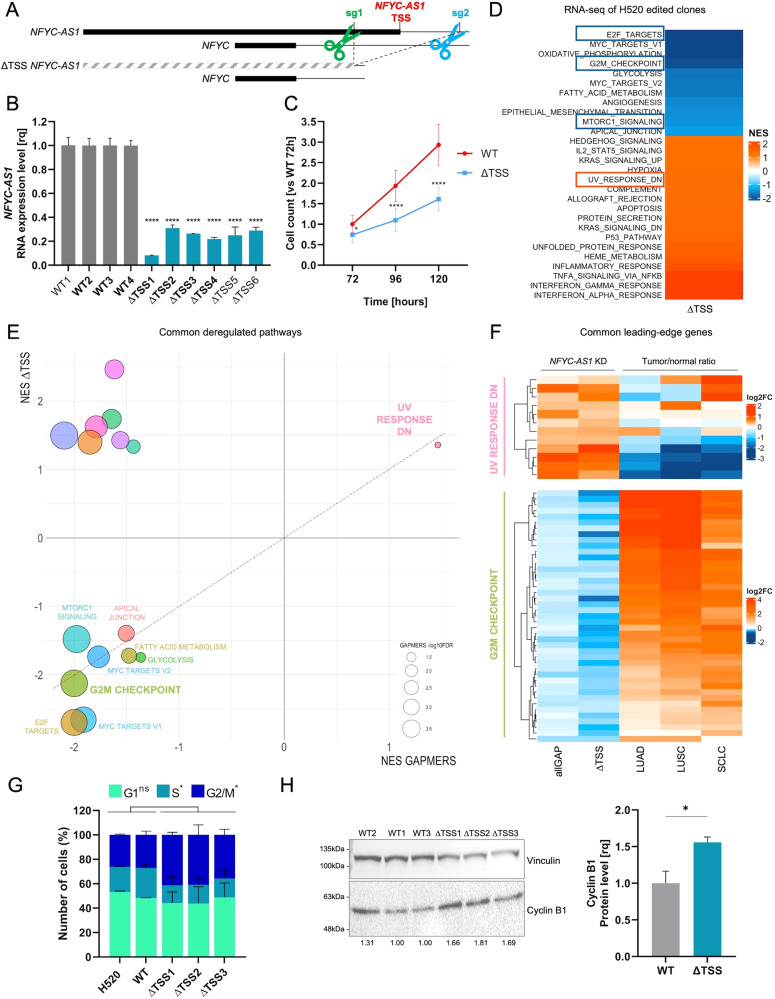
Characterization of *NFYC-AS1* knockout phenotype by CRISPR/Cas9 editing. **A** Schematic representation of the CRISPR/Cas9 strategy employed for *NFYC-AS1* TSS deletion (primers used to check for the deletion are shown in Fig. 3A and PCR results in Supplementary Fig. S5A). **B** Bar plot showing *NFYC-AS1* expression measured through qRT-PCR using primer 4 (Pr4) in four non-deleted (WT) and six deleted (ΔTSS) H520 clones. Data are reported as mean ± se, as from *n* = 3 independent qRT-PCR measurements. Clones subjected to RNA-seq (chosen among those having the most significant down-regulation of *NFYC-AS1*) are indicated in bold. One sample *t*-test *p*-values are reported. **C** Cell counts of WT and ΔTSS H520 clones at 72 h, 96 h and 120 h after plating, normalized against the average cell number in WT clones at 72 h. Data are reported as mean ± se, as from *n* = 3 independent biological replicates. Two-tailed unpaired *t*-test *p*-values are reported. **D** Heatmap of significant NES (FDR < 0.10) of cancer hallmarks (GSEA) for genes differentially expressed in ΔTSS clones compared with WT clones. **E** Comparative bubble plot of significant NES (FDR < 0.10) of cancer hallmarks (GSEA) for differentially expressed genes in Gapmer-treated H520 and in ΔTSS clones. Bubble size is proportional to the FDR (-log_10_FDR) of all Gapmers vs NEG comparison. **F** Heatmap of the fold-change (log_2_FC) of common leading-edge genes of *UV response DN* and *G2/M checkpoint* gene sets in all the Gapmers (vs NEG) and in ΔTSS clones (vs WT) and the relative tumor/normal ratio (expressed as log_2_FC) in LUAD, LUSC (TCGA), and SCLC (GSE60052). **G** Cell cycle analysis by FACS of parental H520 cells, ΔTSS and WT clones. Data are reported as mean ± sd, as from *n* = 2 independent biological replicates. Two-tailed unpaired *t*-test *p*-values are reported. **H** Representative western blot analysis of cyclin B1 in ΔTSS (*n* = 3) and WT (*n* = 3) clones. Vinculin was used as a loading control. Unpaired two-tailed *t*-test *p*-values are reported. *p < 0.05, **p < 0.01, ***p < 0.001, ****p < 0.0001, ns (non-significant).

Given that *NFYC-AS1* knock-down by Gapmers results from RNA cleavage and that asRNAs may also work in a transcription- rather than RNA-dependent manner [3], we attempted to block *NFYC-AS1* transcription through a CRISPR/Cas9-based approach. Since the removal of the whole *NFYC-AS1* sequence would disrupt *NFYC* promoter, we designed two sgRNAs to selectively delete the −independently regulated as from our previous assessments (Fig. 3A)− *NFYC-AS1* TSS from *NFYC* first intron (Fig. 5A) in H520 cells. Several edited heterozygous clones (ΔTSS) were isolated and confirmed to express markedly lower levels of the asRNA as compared to non-edited clones (WT) (Fig. 5B). ΔTSS cells showed impaired cell growth (Fig. 5C), recapitulating the phenotype observed in Gapmer-transfected cells. Similarly, comparison of transcriptional profiles of four independent ΔTSS clones with three independent WT clones revealed significant downregulation of *E2F targets*, *G2/M checkpoint* genes, and *mTORC1 signaling* (Fig. 5D). The most concordant NES among ASO and genetic editing experiments was found for *G2/M checkpoint* and *UV response down* gene sets within down- and upregulated genes, respectively (Fig. 5E). Notably, leading edge genes of both gene sets (Supplementary Table S12) showed inverse patterns of expression in the comparison between tumor and normal samples in LUAD, LUSC and SCLC cohorts (Fig. 5F). In particular, up to 98% of G2/M genes downregulated upon *NFYC-AS1* silencing showed significant upregulation in all cancer histotypes, suggesting that *NFYC-AS1* impacts on cancer-relevant targets. Reactome pathways analyses highlighted that downregulated genes in both comparisons were clearly enriched for gene sets related to mitotic segregation (Supplementary Fig. S5B). In line with these findings, FACS analysis showed that ΔTSS clones tend to accumulate in G2/M cell cycle phase, as compared to parental cells or WT clones, suggesting defects in mitotic progression (Fig. 5G). Accordingly, deleted clones showed increased cyclin B1 protein levels, which is considered a marker for cells stuck in G2/M phase (Fig. 5H).

### Different knockdown approaches shed light on *NFYC-AS1* dual mode of action

**Fig. 6:**
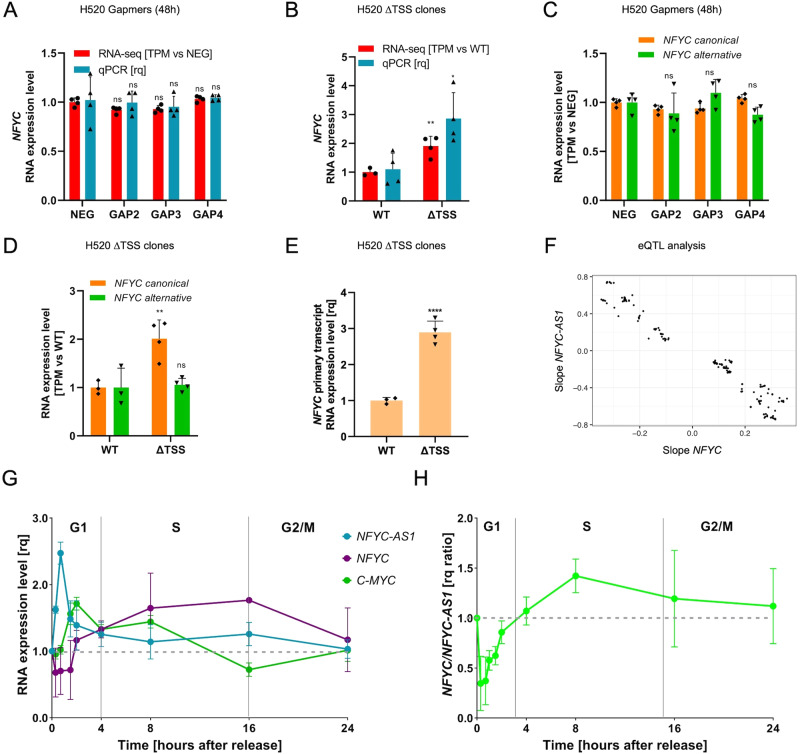
Analysis of *NFYC-AS1* in *cis* function. **A** Bar plot showing *NFYC* expression level (qRT-PCR and RNA-seq) at 48 h after transfection with GAP2-GAP4 in H520 *RB1-*wt cells. Data are NEG-normalized and reported as mean ± sd, as from *n* = 4 independent biological replicates. **B** Bar plot showing *NFYC* expression level (qRT-PCR and RNA-seq) in ΔTSS H520 clones normalized against WT H520 clones. Data are reported as mean ± sd, as from *n* = 4 WT clones and *n* = 4 ΔTSS clones in qRT-PCR and *n* = 3 WT clones and *n* = 4 ΔTSS clones in RNA-seq. **C** Bar plot showing *NFYC* canonical and alternative expression levels (RNA-seq) at 48 h after transfection with GAP2-GAP4 in H520 *RB1*-wt cells. Data are NEG-normalized and reported as mean ± sd, as from *n* = 4 independent biological replicates. **D** Bar plot showing *NFYC* canonical and alternative expression levels (RNA-seq) in ΔTSS H520 clones normalized against WT H520 clones. Data are reported as mean ± sd, as from *n* = 3 WT clones and *n* = 4 ΔTSS clones. **E** Bar plot showing *NFYC* primary transcript expression measured using an intronic primer through qRT-PCR in ΔTSS H520 clones normalized against WT H520 clones. Data are reported as mean ± sd, as from *n* = 3 WT clones and *n* = 4 ΔTSS clones. **F** Scatter plot of effect sizes of eQTLs shared by *NFYC* and *NFYC-AS1*. **G** Time course of *NFYC-AS1*, *NFYC*, and *CMYC* expression levels (qRT-PCR) after re-entry of H520 cells into the cell-cycle. Data are reported as mean ± sd, as from *n* = 3 independent biological replicates. **H** Time course of *NFYC-AS1/NFYC* relative induction ratio after re-entry of H520 cells into the cell-cycle. Data are reported as mean ± sd, as from *n* = 3 independent biological replicates. Two-tailed unpaired *t*-test *p*-values are reported for panels **A**–**E**. deseq2 adjusted *p*-values are reported for panels **A** and **B**. *p < 0.05, **p < 0.01, ***p < 0.001, ****p < 0.0001, ns (non-significant).

Cell growth impairment consequent to *NFYC-AS1* silencing could be compatible with in *cis* interference with its overlapping sense gene *NFYC*, since NF-Y inhibition was shown to trigger proliferation-related defects, including repression of G2/M genes [36]. However, no major effect on *NFYC* expression was found upon Gapmer treatment of H520 cells either at the mRNA (Fig. 6A) or protein level (Supplementary Fig. S6A), regardless of the extent of *NFYC-AS1* repression by the different Gapmers or time points (Supplementary Fig. S6B). The same result was recapitulated in H82 cells treated with Gapmers at either dose (Supplementary Fig. S6C). In contrast, *NFYC* mRNA was significantly upregulated in ΔTSS clones (2 to 3-fold increase), as measured by RNA-seq and qRT-PCR (Fig. 6B), though no substantial changes of protein levels were observed (Supplementary Fig. S6A). When *NFYC* transcripts originating from the two main TSSs were analyzed separately, only those transcribed from the canonical *NFYC* TSS (*i.e*., proximal to *NFYC-AS1*) resulted significantly upregulated in ΔTSS clones, whereas no changes were recorded for those transcribed from the alternative *NFYC* TSS in either clones or Gapmer-transfected cells (Fig. 6C, D). Increase of *NFYC* mRNA in ΔTSS clones was ascribable to enhanced transcription, as evidenced by the analysis of *NFYC* primary transcript (Fig. 6E).

Altogether, these findings would suggest that in *cis* regulation of *NFYC* may rely on transcription-dependent mechanisms. Consistent with this, orthogonal approaches inducing *NFYC-AS1* transcriptional knockdown, such as impairment of NF-Y activity by *NFYB* silencing, resulted in markedly increased *NFYC* levels, mainly due to upregulation of canonical TSS-derived isoforms (Fig. 3B).

Indirect evidence supporting the transcriptional interference existing between *NFYC-AS1* and *NFYC* was obtained by the analysis of expression quantitative trait loci (eQTL). We leveraged significant variant-gene associations in different tissues (GTEx v8) [37] and identified 231 variants that are eQTLs for *NFYC-AS1*. Interestingly, 60.2% were eQTLs also for *NFYC* and all of them consistently showed an opposite effect on *NFYC* expression (Fig. 6F).

To get further validation of the transcriptional interference effect in a physiological setting, we assessed the reciprocal expression of *NFYC-AS1* and *NFYC* during the cell-cycle. We found that, upon re-entry after synchronization, cells correctly progressed through the cell cycle, as shown by modulations in cyclin levels (Supplementary Fig. S6D). *NFYC-AS1* expression peaked in the early G1 phase, similar to the early spike gene *C*-*MYC* [38], then decreased to remain stable across the following cell cycle phases (Fig. 6G). *NFYC* mRNA decreased concomitantly to *NFYC-AS1* induction (Fig. 6G), then started to increase immediately after *NFYC-AS1* peak with reversal of sense/antisense fold-change ratio at the G1/S boundary (Fig. 6H), compatible with a transcriptional interference. Notably, this pattern was recapitulated when cells synchronized in the different cell cycle phases as from *Hao* et al. [39]. (Supplementary Fig. S6E) were analyzed.

**Fig. 7:**
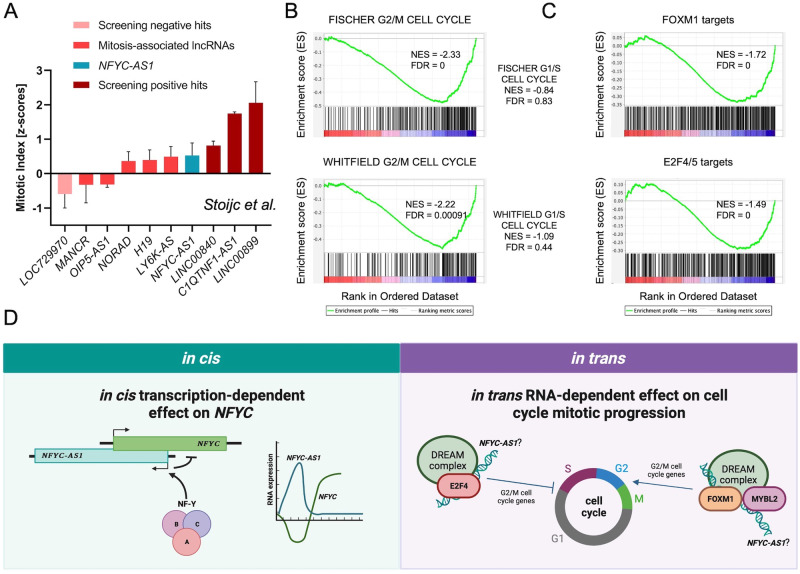
Analysis of *NFYC-AS1* in *trans* function and proposed *NFYC-AS1* dual mode of action. **A** Bar plot showing the average mitotic index z-score ± sd of the two screenings as from *Stojic* et al. [41] for a panel of selected lncRNAs. **B** GSEA enrichment plots of FISCHER G1/S and G2/M CELL CYCLE, and WHITFIELD G1/S and G2/M CELL CYCLE gene sets in genes modulated upon *NFYC-AS1* Gapmer-silencing. NES and FDR values are indicated in each plot. **C** GSEA enrichment plots of FOXM1 and E2F4 bound genes (defined as described in Supplementary Materials and Methods section) in genes modulated upon *NFYC-AS1* Gapmer-silencing. NES and FDR values are indicated in each plot. **D** Schematic representation of the in *cis* and in *trans* possible effects, mechanism(s) of action and biological role of *NFYC-AS1*.

The observation that cell growth decline invariably occurred upon *NFYC-AS1* silencing with both Gapmers and TSS deletion suggests that this phenotype is i) at least in part *NFYC*-independent and ii) mediated by RNA- rather than transcription-dependent mechanisms. In support of the first hypothesis, TransCistor tool [40] predicted a predominantly in *trans* function from both Gapmer and ΔTSS RNA-seq data (Supplementary Fig. S6F). Independent validation of *NFYC-AS1* RNA-dependent role in the cell cycle was obtained by interrogating an RNAi-based screening for lncRNAs involved in cell division [41]. When analyzing the mitotic index (*i.e*., number of cells stuck in mitosis), z-score of *NFYC-AS1* appeared in line with (or superior to) that of other lncRNAs demonstrated to regulate mitotic progression, such as *NORAD1* [42] or *LY6K-AS* [43] (Fig. 7A).

Consistent with this, genes repressed by Gapmer treatment (the optimum to selectively visualize in *trans*-mediated effects) were significantly enriched in G2/M and not in G1/S genes (Fig. 7B) and displayed a highly significant enrichment of TF binding sites (defined based on ChIP-seq signal and presence of consensus motif in the promoter) of complexes involved in G1/S gene repression (*i.e*., E2F4/5-DREAM) and G2/M gene activation (*i.e*., MYBL2, FOXM1) [44], with moderate or no enrichment for activator E2Fs or NF-Y, respectively (Fig. 7C and Supplementary Fig. S6G).

## Discussion

The identification of asRNAs that could serve as molecular targets in cancer therapy requires the careful study of their expression in tumors and association with molecular/clinical features, as well as a deep understanding of their function. Here, we showed that *NFYC-AS1* is an asRNA overexpressed pancancer, with striking upregulation in the very aggressive *RB1*-mut tumors. Significant discriminative power in distinguishing tumor from normal tissues as well as molecular subtypes (*RB1*-mut vs -wt) may suggest *NFYC-AS1* as a potential cancer biomarker.

We found that *NFYC-AS1* depletion by either ASOs or CRISPR/Cas9 results in cell growth impairment in both *RB1*-wt and -mut cancer cells, mainly due to downregulation of G2/M cell cycle phase genes. The cancer-specific *NFYC-AS1* upregulation, which can be partly explained by transcriptional activation by NF-Y or E2Fs as a consequence of *RB1* mutations, may indeed exacerbate cell proliferation by facilitating mitotic progression.

From a therapeutic perspective, *NFYC-AS1* knockdown simultaneously repressed genes that represent vulnerabilities of all lung cancer histotypes. Consistent with this model, *NFYC-AS1* silencing impairs cell growth in both LUSC and SCLC cells, as reported in this study, and induces apoptosis in LUAD cells, as shown in the work by Song and colleagues [19]. Moreover, *NFYC-AS1* knockdown inhibited genes that are synthetic lethal in *RB1*-mut cells, suggesting a therapeutic potential in the very aggressive *RB1*-mut tumors, for which current treatments are often unsuccessful [45]. Moreover, defects in mitotic progression arising from *NFYC-AS1* depletion may create new vulnerabilities, which can be particularly deleterious in *RB1*-defective cancers, as already shown for Aurora B kinase inhibitors [35]. At this stage, we cannot conclude whether the apparently lower sensitivity of H82 cells to *NFYC-AS1* knockdown as compared to H520 cells is the result of an intrinsically higher resistance of *RB1*-mut cells or is rather related to the different cell growth pattern. In this regard, studies using isogenic models of *RB1*-mut and -wt cells are warranted to address this point.

From a mechanistic point of view, our data suggest that *NFYC-AS1* may have a dual mode of action (Fig. 7D): it represses its sense gene *NFYC* in *cis* in a transcription-dependent manner, while it regulates G2/M cell cycle phase genes in *trans* in a transcription- and apparently *NFYC*-independent manner. In this regard, being completely in line with those of other lncRNAs, the observed levels of conservation of *NFYC-AS1* locus would support the relevance of sequence-based mechanisms. It is not surprising that asRNAs overlapping with sense genes may also (or even only) work in *trans*, as shown for *GNG12-AS1* which represses its sense gene in *cis* via transcriptional interference, while it regulates other genes involved in cell proliferation and migration in *trans* through an RNA-dependent mechanism [46].

Regarding *NFYC-AS1* in *cis* activity, we conclude it is mediated by a transcription-dependent mechanism as it becomes evident only when asRNA transcription is affected (*e.g*., CRISPR/Cas9-editing, NF-Y depletion, or cell cycle re-entry assay). Specifically, *NFYC-AS1* seems to antagonize *NFYC* expression, likely through transcriptional interference. An opposite expression pattern between the two transcripts is also evident in different human tumors as compared to matched healthy tissues and in eQTL analysis. At this stage, mechanisms relying on *NFYC-AS1* transcript, such as the formation of R-loops with DNA elements in *NFYC* promoter [47], pairing with *NFYC* pre-mRNA [48], or interaction with protein factors are unlikely to occur, as the in *cis* effect was not recapitulated by any of the ASOs spanning the whole asRNA sequence. Transcriptional overlap mechanism proposed for *Airn* [49] is also improbable, as cleavage of *NFYC-AS1* just downstream of its TSS by GAP2 does not relieve repression on *NFYC*. Only mechanisms strictly associated with the act of transcription per se, such as RNA pol-II dislodgement/occlusion/collision/roadblock events (reviewed in *Zhao* et al. [11]), the creation of a repressive chromatin state by epigenetic histone modifications [10, 50] or nucleosome re-positioning [51], might be compatible with our findings. In light of our ΔTSS model, DNA-dependent or topological effects, as shown for *HASTER* promoter on *HNF1A* expression [52], cannot be completely excluded. However, approaches commonly used to manipulate endogenous lncRNA transcription, such as CRISPR-activation or inhibition, are not applicable to *NFYC-AS1*, as unintended effects on the adjacent *NFYC* TSS are likely to bias results [53]. Biologically, *NFYC-AS1* may serve to finely tune *NFYC* transcription timing at the beginning of the cell cycle and/or contribute to the negative feedback loop existing between *NFYB* and *NFYC* [54] (Fig. 7D). It was indeed shown that *NFYB* knockdown results in increased *NFYC* mRNA levels (also evident in Fig. 3B), which could be explained by direct activation of *NFYC-AS1* by NF-Y and subsequent impaired *NFYC* transcription. Observed patterns of sequence conservation and stretches of MIR at the *NFYC-AS1* locus are suggestive of a mammalian evolutionary origin, with similar considerations applying to the canonical *NFYC* TSS. This arrangement might represent an additional layer of regulation of *NFYC* transcription, which instead should lack in other species having only the ancestral TSS, regulated directly by NF-Y.

Regarding *NFYC-AS1* in *trans* mode of action, it appears to converge on the regulation of G2/M cell cycle phase genes, in trend with the mitotic arrest phenotype observed in ΔTSS cells and in Stojic’s RNAi-based screening [41]. Further investigation will be required to define i) which among these genes are direct or indirect targets, ii) which is the exact amount of the asRNA in the cell (*i.e*. copies per cell) and its subnuclear distribution, and iii) whether RNA-dependent effects are mediated by *NFYC-AS1* sequence or 3D structure, as well as the nature of its interactors. In this regard, the insertion of repeated elements, such as the presence of *SINE*/*Alu* at *NFYC-AS1* 3’ end (Fig. 3A), may have endowed it with a novel in *trans* function. Transposable elements have been proven to act as functional modules for a number of lncRNAs [7, 55], including *MIR205HG*, which we showed to use an *Alu* element to physically interact with *Alu* elements in target gene promoters [56, 57]. *NFYC-AS1* may interact with direct targets through RNA/RNA pairing to affect mRNA processing/stability or RNA/DNA interaction with their regulatory regions, in the form of triplexes or R-loops. Indirect targets may instead be regulated via an intermediate factor (*e.g*. TF or RBP), itself physically bound/stabilized/sequestered by *NFYC-AS1*. LncRNAs can indeed directly interact with TFs, as shown for *PANDA* that acts as a molecular decoy for NF-YA, thereby titrating the TF away from its targets [58]. LncRNAs can also function as RNA guides to facilitate TF interaction with specific genes, as is the case of *SLC16A1-AS1*, which simultaneously acts as guide and chaperone/coactivator for E2F1 [59]. All the scenarios could be interrogated to dissect *NFYC-AS1* mechanism of action in *trans*. Speculatively speaking, *NFYC-AS1* might interact with E2F4/5 for the correct assembly/stability/activity of the DREAM complex to silence G2/M cell cycle genes during G1/S phase or sequester FOXM1 in G1/S phase until its expression decreases releasing FOXM1 to allow activation of G2/M genes (Fig. 7D). Given that NF-Y cooperates with these TFs in the regulation of common targets [60], *NFYC-AS1* may also tune NF-Y function by altering TF multicomplex assembly, cooperativity, and genome-wide co-occupancy, especially on selected subsets of targets.

A hypothesis conciliating *NFYC-AS1* dual modes of action could be that NF-Y (and/or RB1/E2F axis) induces *NFYC-AS1* expression to i) finely tune *NFYC* transcription in *cis* in a negative feedback loop circuit and ii) simultaneously in *trans* stimulate the activity of known NF-Y partners on G2/M-specific genes via chaperoning DREAM/MYBL2/FOXM1 activity (Fig. 7D). Intriguing could be to evaluate whether the in *cis* fine-tuning effect may prevail in normal cells at low *NFYC-AS1* concentrations, whereas the in *trans* effect may become predominant in tumor cells to support excessive proliferation and mitotic progression.

Overall, we showed that *NFYC-AS1* is an optimal candidate to be evaluated in the long term as a new target entity in different cancer types, including the very aggressive *RB1*-mut tumors. Moreover, the dissection of *NFYC-AS1* mechanism of action and the identification of its interactors may provide a rich environment of actionable protein-coding genes that can be targeted with already existing drugs.

### Supplementary information

Original Data 1

Original Data 2

Supplementary Figures and Methods

Supplementary Tables

reproducibility checklist

## Data Availability

All data used in this work can be acquired from the TCGA database (http://cancergenome.nih.gov/), ENCODE database (http://encodeproject.org/), CCLE database (http://sites.broadinstitute.org/ccle), Gene Expression Omnibus (GEO) datasets (https://www.ncbi.nlm.nih.gov/geo/), GTEx project (https://gtexportal.org/home/). Raw and processed RNA-seq data of H520 cells transfected with Gapmer ASOs or deleted for *NFYC-AS1* TSS have been deposited at GEO under accession number GSE240468 and are available from the corresponding author upon reasonable request.
