# Supplementary material for: The pancancer overexpressed NFYC Antisense 1 controls cell cycle mitotic progression through in cis and in trans modes of action
Source: Cell Death Dis. 2024 Mar 11;15(3):206. doi: 10.1038/s41419-024-06576-y (PMC10928104; doi:10.1038/s41419-024-06576-y)
Supplement: Supplementary file 2 — Original Data 2 [file 41419_2024_6576_MOESM2_ESM.pdf]

ORIGINAL DATA 2

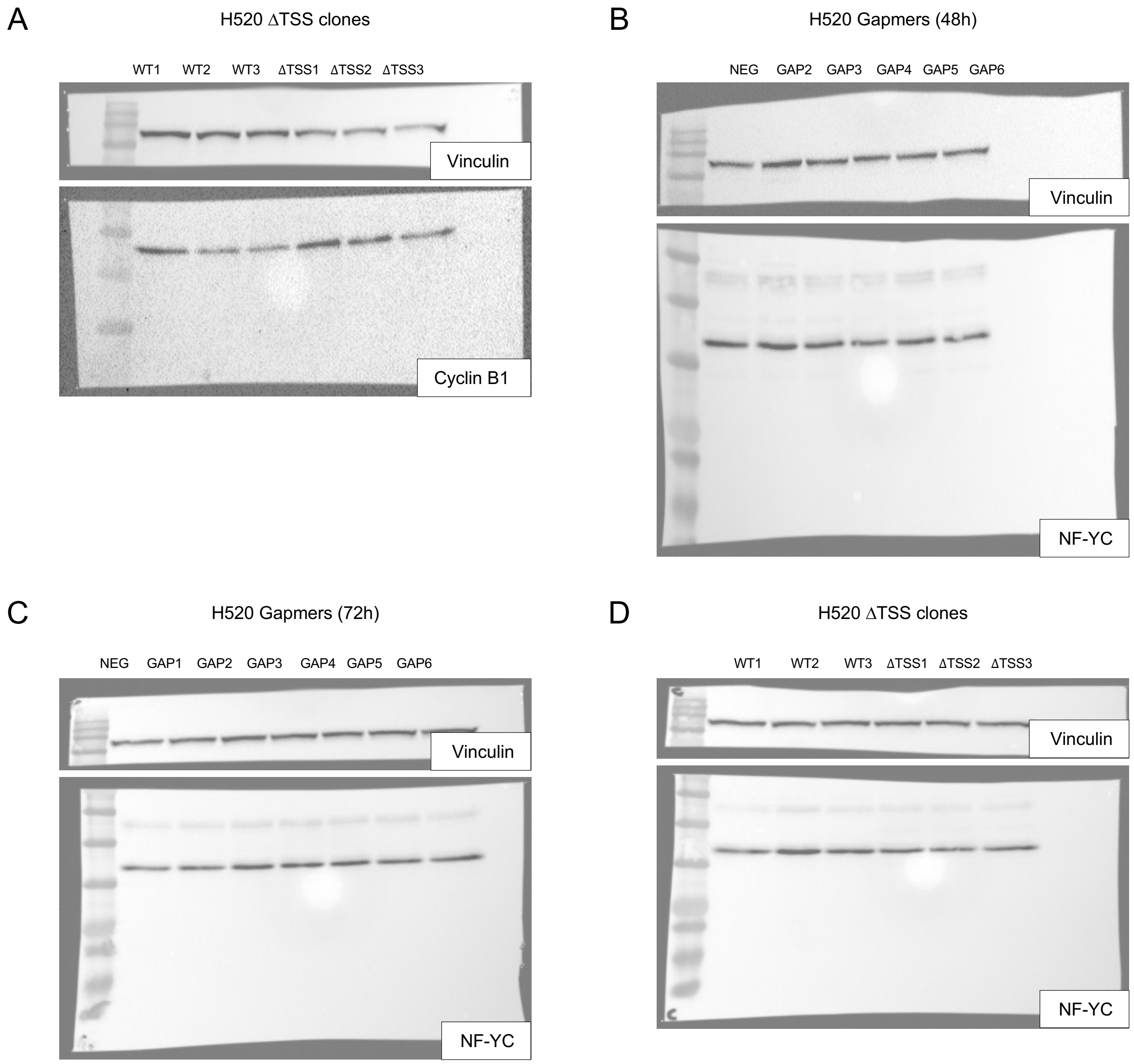

Full length uncropped original western blots. **A** Representative western blot analysis of cyclin B1 in WT and ΔTSS H520 clones (relative to Figure 5H). **B** Representative western blot analysis of NF-YC in H520 cells at 48h or **C** 72h after transfection with Gapmers (GAP1-GAP6) and NEG, and **D** in WT and ΔTSS H520 clones (relative to Supplementary Figure S6A). Vinculin was used as loading control.
