# Supplementary material for: The pancancer overexpressed NFYC Antisense 1 controls cell cycle mitotic progression through in cis and in trans modes of action
Source: Cell Death Dis. 2024 Mar 11;15(3):206. doi: 10.1038/s41419-024-06576-y (PMC10928104; doi:10.1038/s41419-024-06576-y)
Supplement: Supplementary file 3 — Supplementary Figures and Methods [file 41419_2024_6576_MOESM3_ESM.pdf]

SUPPLEMENTARY FIGURES

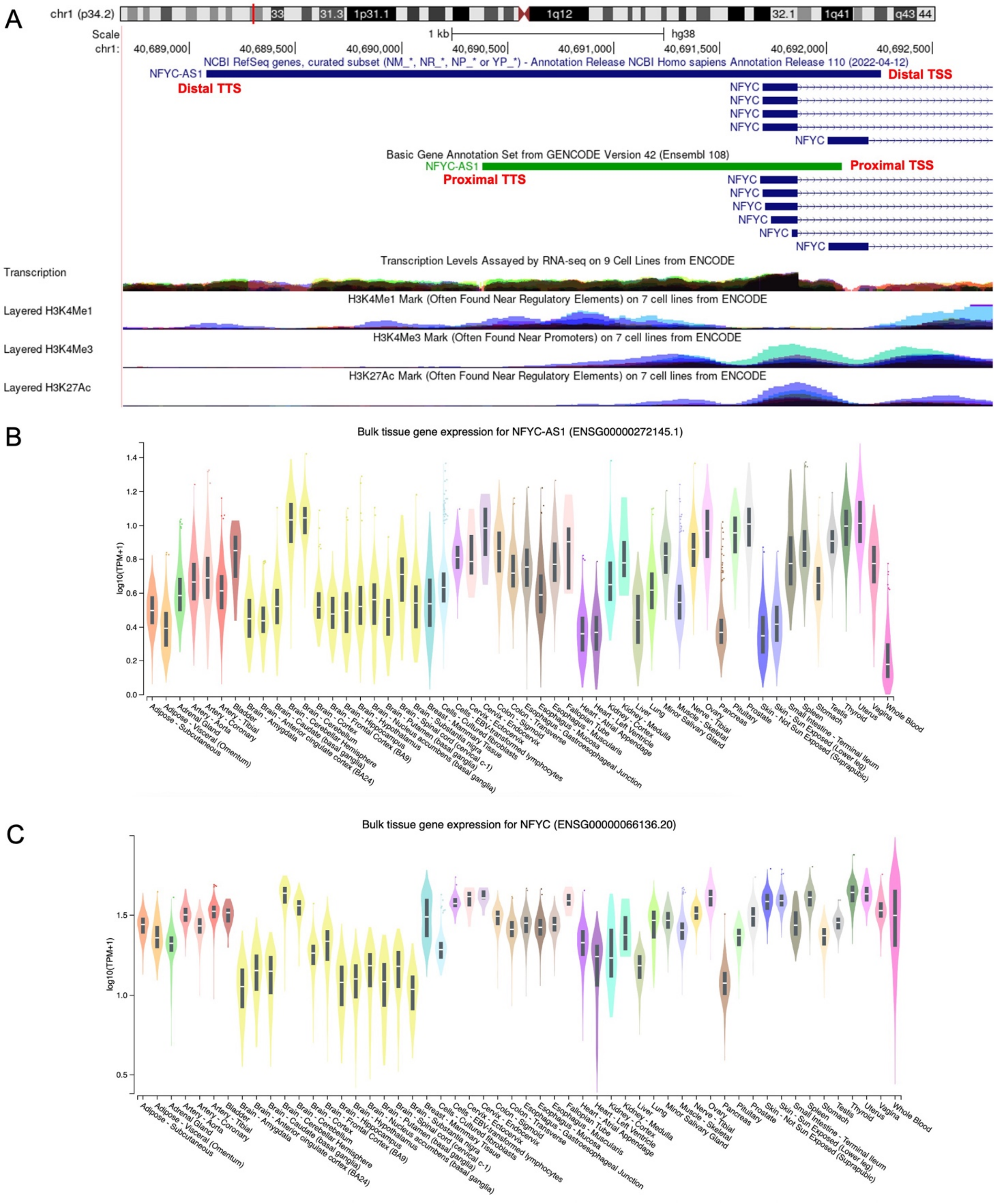

Supplementary Figure S1: ***Current annotation of NFYC-AS1.*** **A** *NFYC(-AS1)* locus at chr1p34.2 as from UCSC Genome Browser (GRCh38/hg38 assembly). The NCBI RefSeq and GENCODE V42 annotations together with the transcription activity, H3K4Me1, H3K4Me3, and H3K27Ac histone

modifications (ENCODE) are reported. **B** Violin plots of *NFYC-AS1* and **C** *NFYC* expression across GTEx human tissues (Data Source: GTEx Analysis Release V8 (1)). Boxes show median and interquartile ranges.

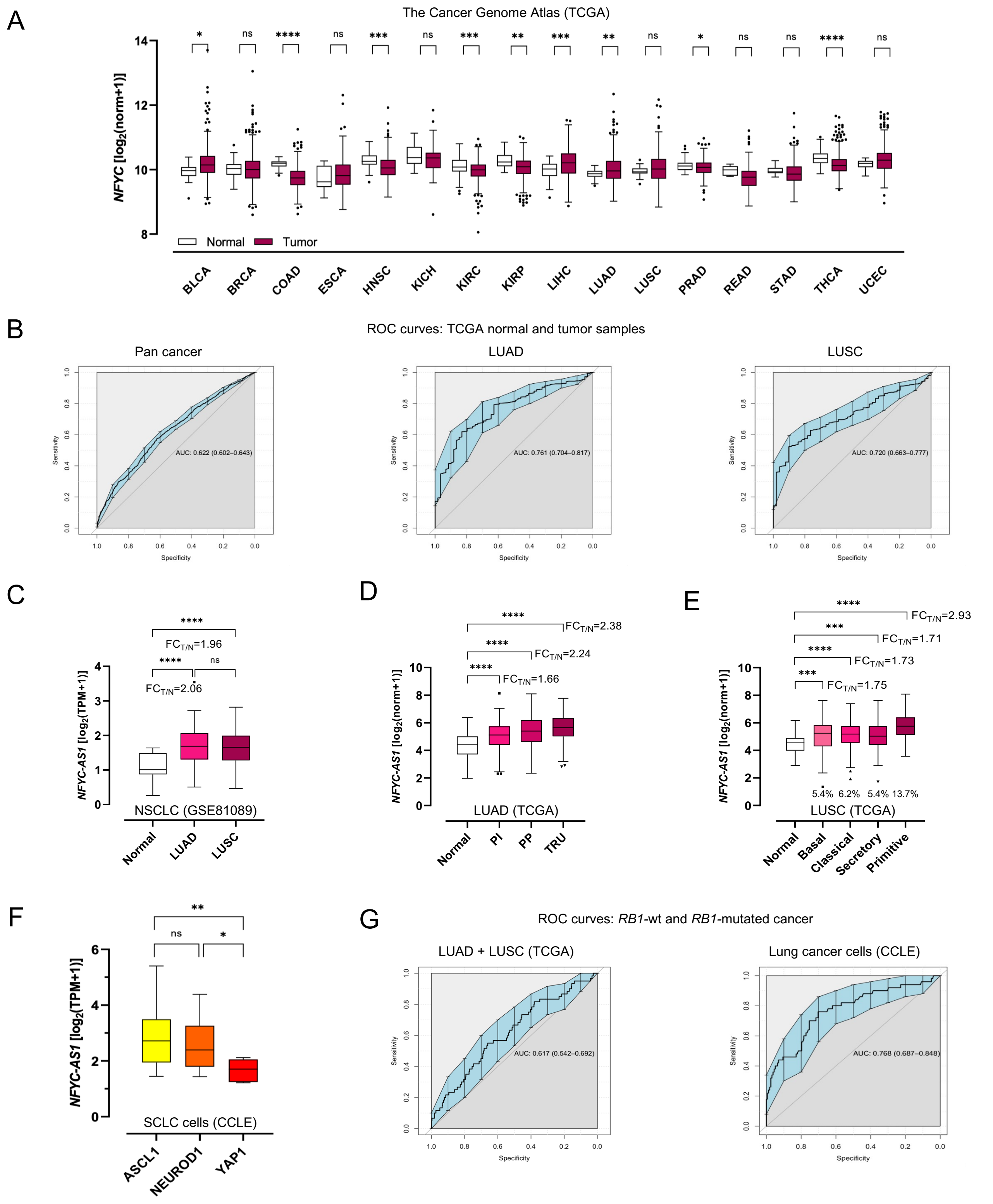

Supplementary Figure S2: *NFYC-AS1 expression in tumor tissues and cells*. A Boxplots of differential expression of *NFYC* in normal and tumor tissues (TCGA (2)). **B** ROC curves to test normal-tumor classification according to *NFYC-AS1* expression in different cancer types (pancancer), LUAD or LUSC (TCGA). The area under the curve (AUC) and the confidence interval are reported in the figure. For a good classifier: 0.5 < AUC <1. **C** Boxplots of *NFYC-AS1* expression level in normal tissues and in LUAD and LUSC NSCLC histotypes (GSE81089). **D** Boxplots of *NFYC-AS1* expression level in normal

tissues and in the different LUAD and **E** LUSC molecular subtypes (TCGA). Frequency of *RB1* mutation for LUSC molecular subtypes (TCGA) is reported on the graph. **F** Boxplots of *NFYC-AS1* expression level in SCLC cell lines classified by their molecular subtype (CCLE (3)). **G** ROC curves to test *RB1*-wildtype/-mutated classification according to *NFYC-AS1* expression in different NSCLC tissues (TCGA) and lung cancer cell lines (CCLE). The area under the curve (AUC) and the confidence interval are reported in the figure. For a good classifier: 0.5 < AUC <1.

Throughout the figure, the gene expression level is expressed as logarithm in base 2 of the normalized counts (norm) or TPM plus one [$\log_2$(norm or TPM+1)]. The tumor-normal fold-change ($FC_{T/N}$) is calculated as the ratio between the average normalized counts or TPM for *NFYC-AS1* in tumors and in normal tissues. *p*-values of the two-tailed unpaired *t*-test are reported, *p<0.05, **p<0.01, ***p<0.001, ****p<0.0001, ns (non-significant). [PI (proximal inflammatory), PP (proximal proliferative), TRU (terminal respiratory unit)].

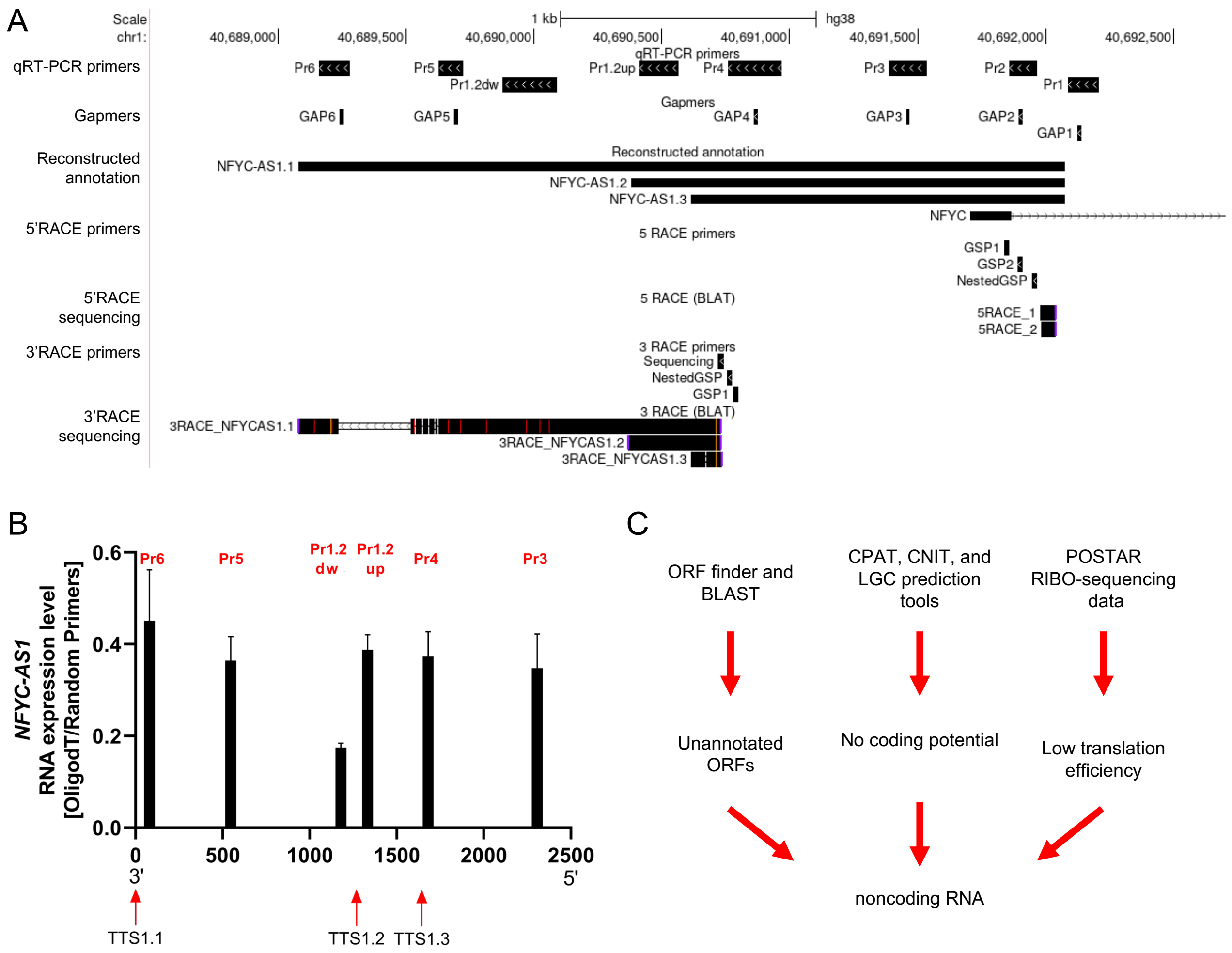

Supplementary Figure S3: *NFYC-AS1 transcript reannotation*. **A** *NFYC(-AS1)* locus at chr1p34.2 as from UCSC Genome Browser (GRCh38/hg38 assembly). On the top, the design of qRT-PCR primers and Gapmers is shown together with the *NFYC-AS1* reconstructed annotation. On the bottom, the design of the primers and the sequencing output is reported for both 5'RACE and 3'RACE assays. **B** Bar plot showing the ratio of *NFYC-AS1* expression as measured from cDNA retrotranscribed using random oligo-dT primers versus random primers. qRT-PCR was conducted using different primers (indicated on top of the bars) spanning the whole *NFYC-AS1* sequence. Data are reported as mean ± sd as from n=3 independent biological replicates. **C** Schematic workflow for the study of the noncoding potential of *NFYC-AS1* using ORF Finder (4), CPAT (5), CNIT (6), LGC (7), and POSTAR (8) tools.

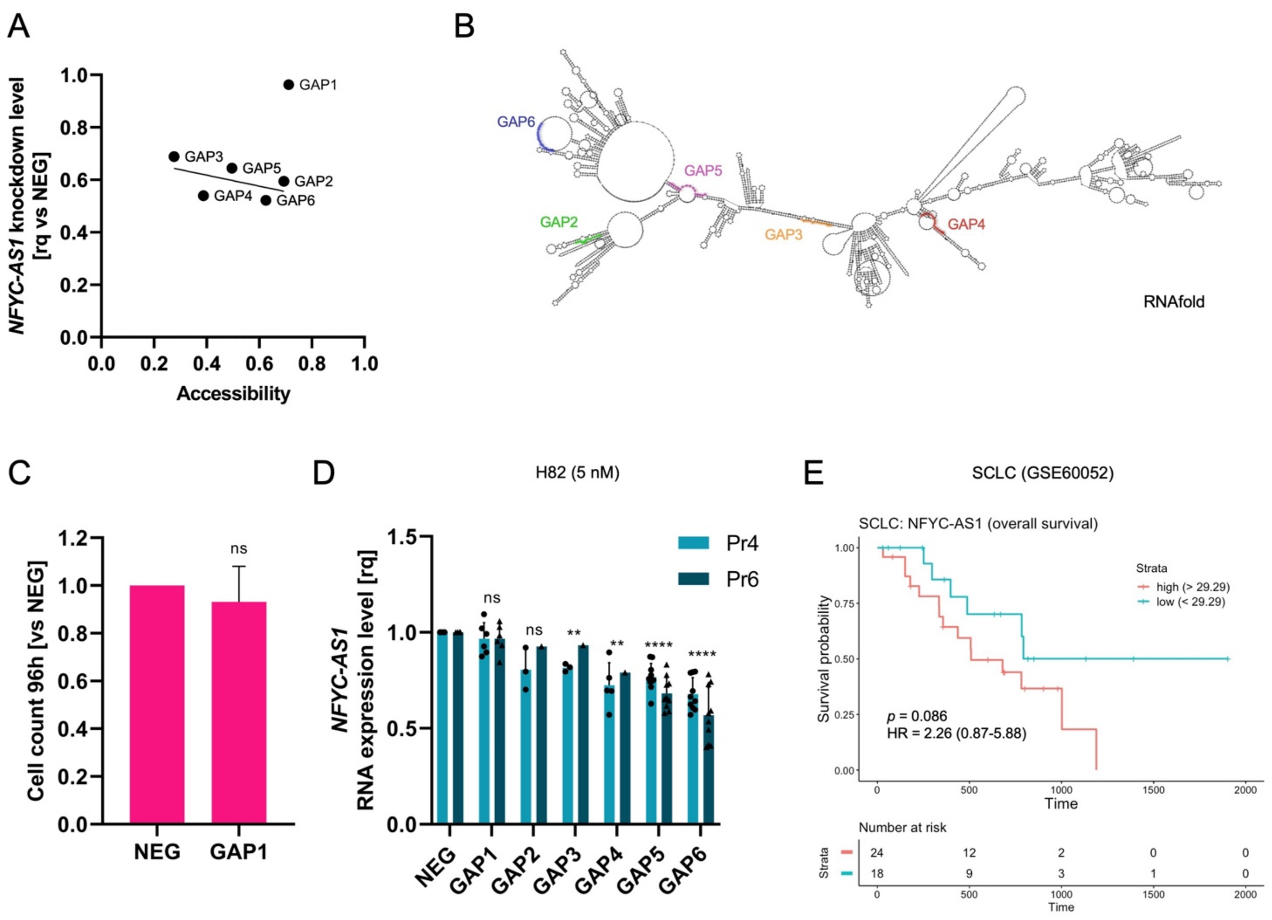

Supplementary Figure S4: ***Characterization of NFYC-AS1 knockdown phenotype by Gapmer ASOs***. **A** Relation between the estimated accessibility of each Gapmer-targeted region calculated as the average of the accessibility percentage of the single bases belonging to the *NFYC-AS1* portions targeted by the different Gapmers (GAP1-GAP6) (as from S-fold(9)) and the *NFYC-AS1* knockdown level reached with the different Gapmers in H520 *RB1* wt cells. The *NFYC-AS1* accessibility analysis was performed using our reconstructed annotation to estimate the accessibility of each Gapmer-targeted region for GAP2-GAP6 and the NCBI RefSeq annotation for GAP1. **B** *NFYC-AS1* predicted secondary structure (as from RNAfold (10)); the different Gapmer-targeted regions are highlighted in the structure. Our reconstructed annotation was used for this analysis. **C** Bar plot showing the total cell count after *NFYC-AS1* knockdown with 5 nM GAP1 used at 96h normalized against NEG in H520 *RB1* wt cells. Data are reported as mean ± sd, as from n=3 independent biological replicates. *p*-values of the two-tailed unpaired *t*-test are reported. **D** Bar plot showing *NFYC-AS1* expression measured through qRT-PCR using primer 4 (Pr4) or primer 6 (Pr6) at 48 hours after transfection with all six Gapmers (GAP1-GAP6), normalized against NEG in H82 *RB1* mut cells. Gapmers were used at a final concentration of 5 nM. Data are reported as mean ± sd as from multiple independent biological replicates (n indicated in Supplementary Table S11). *p*-values of the one sample *t*-test are reported. **E** Overall survival curve with stratification according to Cutoff Finder (11) -determined threshold (29.29) for *NFYC-AS1* in SCLC patients (GSE60052). *p*-value calculated using the log-rank test and the hazard risk (HR) are reported in the figure. *$p<0.05$, **$p<0.01$, ***$p<0.001$, ****$p<0.0001$, ns (non-significant).

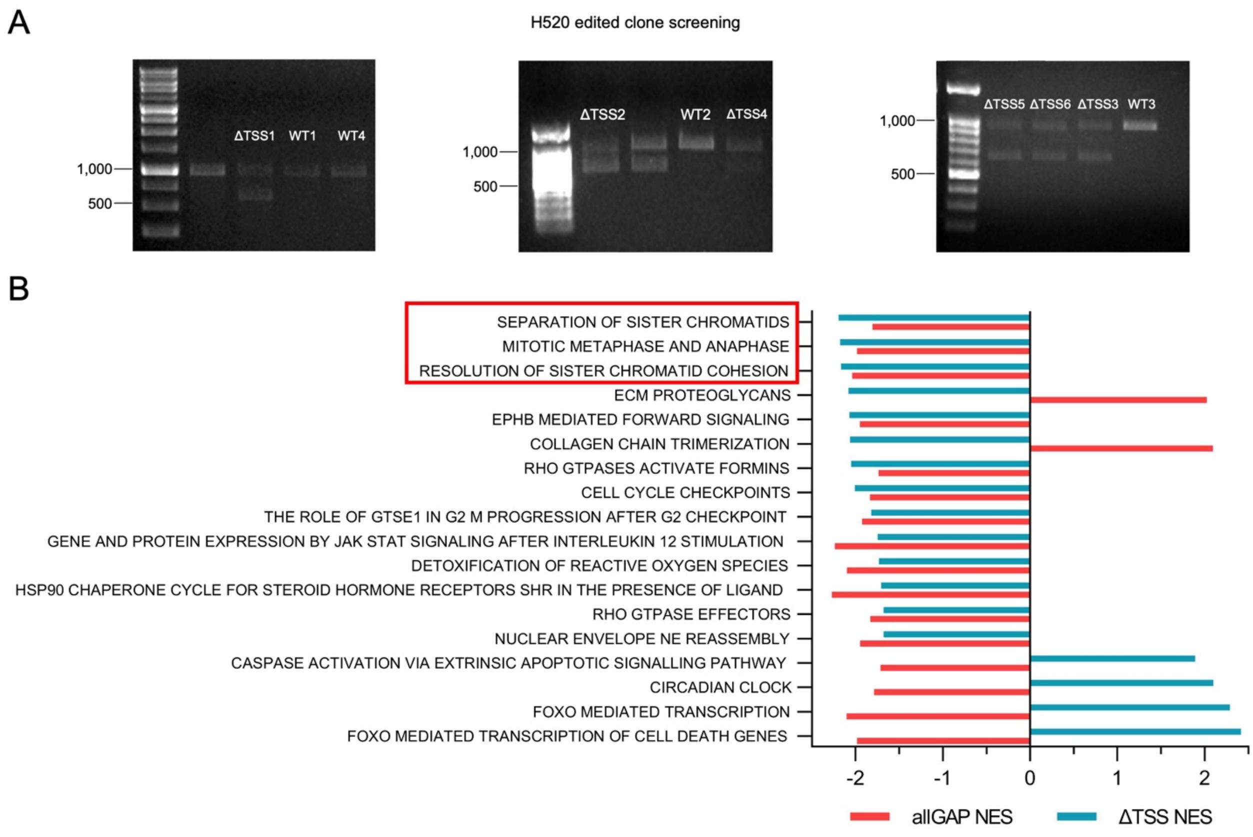

Supplementary Figure S5: ***Characterization of NFYC-AS1 knockout phenotype by CRISPR/Cas9 editing***. **A** Electrophoretic gel of the PCR products amplified with the genomic PCR primers in WT and ΔTSS H520 clones. **B** Comparative bar plot of significant NES (FDR<0.05) of GSEA (12) C2 Reactome gene sets run on RNAseq of Gapmer-treated H520 and of ΔTSS clones.

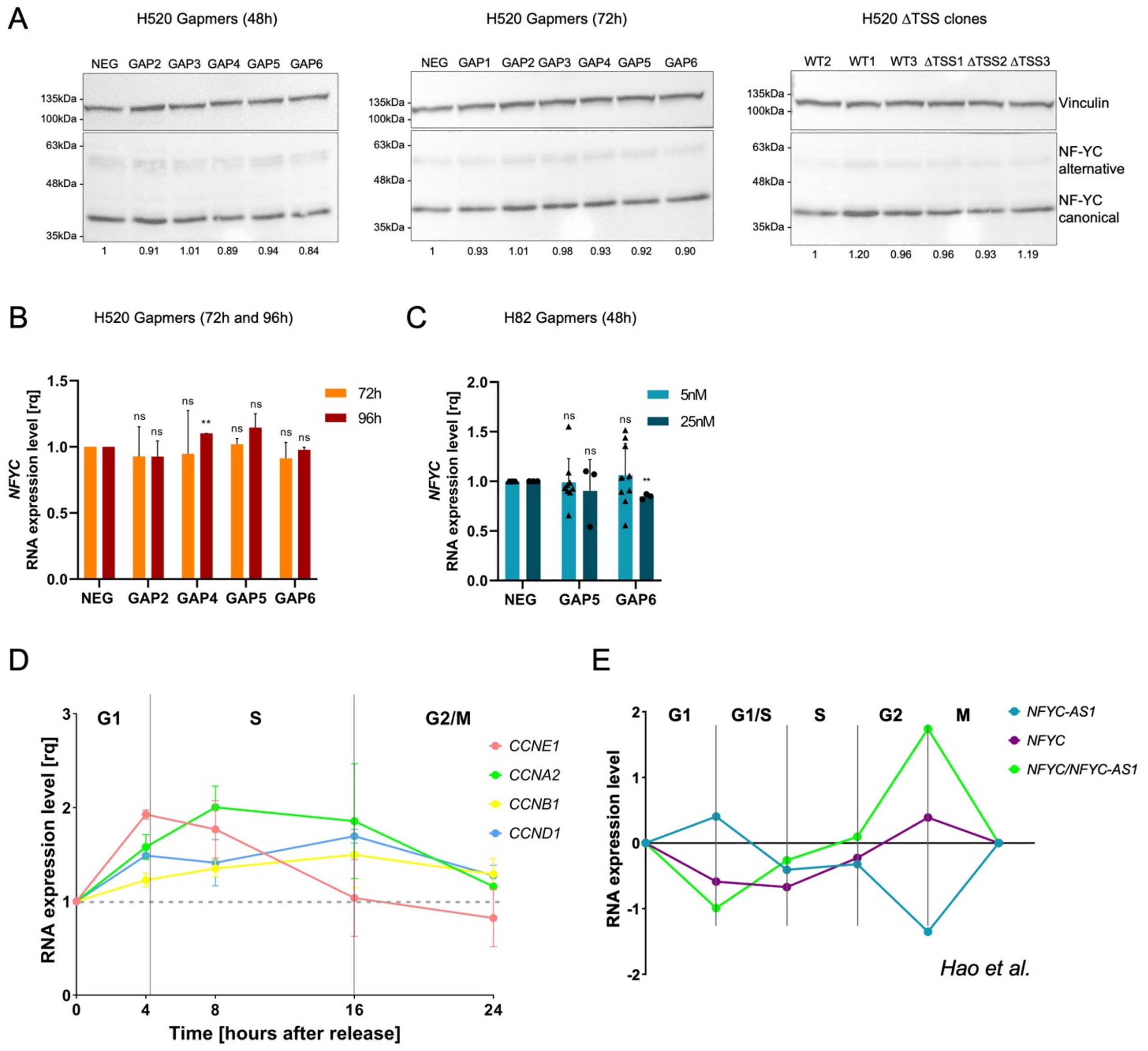

F

	TransCistor
GAP2 vs NEG	*in trans (padj < 0.01)*
GAP3 vs NEG	*in trans (padj < 0.01)*
GAP4 vs NEG	*in trans (padj < 0.01)*
ΔTSS vs WT	*in trans (pval < 0.01)*

G

NAME	NES	NOM p-val	FDR q-val
FOXM1 TARGETS	-1.717	0.000	0.000
E2F4/5 TARGETS	-1.489	0.002	0.003
MYBL2 TARGETS	-1.471	0.000	0.005
E2F1/2/3 TARGETS	-1.201	0.087	0.058
NF-Y TARGETS	-1.139	0.126	0.118

Supplementary Figure S6: ***Analysis of NFYC-AS1 in cis and in trans mechanism of action***. **A** Representative western blot analysis of NF-YC in H520 cells at 48h and 72h after transfection with Gapmers (GAP1-GAP6) and NEG, and in WT and ΔTSS H520 clones. Vinculin was used as loading control. Quantification of NF-YC canonical band intensities through ImageJ software is normalized against vinculin band intensities and against the control (NEG or WT) and is reported below. **B** Bar plot showing *NFYC* expression measured through qRT-PCR at 72h and 96h after transfection with GAP2, GAP4, GAP5, and GAP6 normalized against NEG in H520 *RB1* wt cells. Data are reported as mean ± sd, as from n=3 independent biological replicates. *p*-values of the one sample *t*-test are

reported. **C** Bar plot showing *NFYC* expression measured through qRT-PCR at 48h after transfection with GAP5 and GAP6 at 5 nM and 25 nM concentration, normalized against NEG in H82 *RB1* mut cells. Data are reported as mean ± sd (n indicated in Supplementary Table S11). *p*-values of the one sample *t*-test are reported. **D** Time course of the expression level of cyclins (*CCNE1*, *CCNA2*, *CCNB1* and *CCND1*) after re-entry of H520 cells into the cell-cycle, as measured through qRT-PCR. Data are reported as mean ± sd, as from n=3 independent biological replicates. **E** Time course of the expression level of *NFYC-AS1, NFYC* and *NFYC/NFYC-AS1* induction ratio through the different cell cycle phases as from *Hao et al.* (13), expressed as $\log_2$FC versus time 0. **F** TransCistor (14) output for Gapmers (GAP2-GAP4) versus NEG and ΔTSS H520 clones versus WT H520 clones. **G** Table reporting NES and FDR values of GSEA for FOXM1, E2F4/5, MYBL2, E2F1/2/3, and NF-Y bound genes (defined as described in Supplementary Material and Methods section) in genes modulated upon *NFYC-AS1* silencing by Gapmers. **p*<0.05, ***p*<0.01, ****p*<0.001, *****p*<0.0001, ns (non-significant).

SUPPLEMENTARY MATERIALS AND METHODS

Analysis of *NFYC-AS1* expression in publicly available datasets

NFYC-AS1 expression in normal tissues was studied by the investigation of the Genotype-Tissue Expression (GTEx) portal (1).

NFYC-AS1 expression in tumor tissues was analyzed in The Cancer Genome Atlas (TCGA) (2), considering only tissues with at least 10 normal and 10 tumor samples. Normalized TCGA data were downloaded from firebrowse webpage (http://firebrowse.org/). As of November 2020, RNA-sequencing data were available for 576 LUAD samples, including 517 primary tumor samples and 59 non-tumor (normal) lung tissues, and for 552 LUSC samples, including 501 primary tumor samples and 51 normal lung tissues. Metadata were also downloaded with all the information related to the molecular subtype and the mutational status of each sample. The gene expression level was expressed as logarithm in base 2 of the normalized counts (norm) plus one [$\log_2$(norm+1)]. The tumor-normal fold-change ($FC_{T/N}$) was calculated as the ratio between the average normalized counts for *NFYC-AS1* in tumors and in normal tissues. Instead, the *NFYC-AS1*/*NFYC* ratio was calculated as the ratio between the normalized count for *NFYC-AS1* and for *NFYC* in each sample and then averaged.

FASTQ files of the NSCLC dataset (GSE81089), SCLC dataset (GSE60052), and reprogrammed epithelial cells to neuroendocrine small cell (GSE118206) were retrieved using the fastq-dump utility of the SRA toolkit 2.9.4 version (https://sra-explorer.info/). Gene intensities were computed using RSEM-1.3.1 software using NCBI RefSeq (GRCh37/hg19) as a reference to get the transcript per million (TPM) of genes. The gene expression level was expressed as logarithm in base 2 of the TPM plus one [$\log_2$(TPM+1)]. The tumor-normal fold-change ($FC_{T/N}$) was calculated as the ratio between the average TPM for *NFYC-AS1* in tumors and in normal tissues. The NSCLC dataset collects RNA-sequencing data of 19 normal samples and 198 NSCLC samples, which were classified by performing Pearson correlation of each sample with a previously validated 42-genes-classifier (15). Samples with correlation values ranging from -0.17 to 0.17 were not further classified and were not considered, while values above 0.17 were predicted to be LUAD and values below -0.17 were predicted to be LUSC. This allowed the identification of 91 LUAD and 77 LUSC samples. Instead, the SCLC dataset contains 79 SCLC tumors and 7 normal tissues.

Publicly available data for 1305 cell lines derived from different tumors retrieved from the Cancer Cell Line Encyclopedia (CCLE) (3) were downloaded from Depmap portal (version 20Q4, https://depmap.org/portal/). Metadata were also downloaded with all the information related to the histotype of origin and the mutational status for each cell line. The gene expression level was expressed as logarithm in base 2 of the TPM plus one [$\log_2$(TPM+1)]. The mutated-wildtype fold-change ($FC_{mut/wt}$) was calculated as the ratio between the average TPM for *NFYC-AS1* in presence and absence of the mutations. Instead, the *NFYC-AS1*/*NFYC* was calculated as the ratio between the normalized count for *NFYC-AS1* and for *NFYC*. SCLC cell lines were classified into NEUROD1, ASCL1 and YAP1 molecular subtypes according to *Ireland et al.* (16).

Receiver Operating Characteristic (ROC) curves were computed using the pROC R package to test the performance of *NFYC-AS1* expression as a binary classifier of normal and tumor tissues in different cancer types, NSCLC, LUAD, and LUSC (TCGA) and of *RB1*-wildtype and *RB1*-mutated LUAD and LUSC tissues (TCGA), and lung cancer cell lines (CCLE). The area under the curve (AUC) is computed using the *roc* function from the pROC R package. For a good binary classifier an AUC > 0.5 is expected.

Nucleus-cytoplasm fractionation

For nucleus-cytoplasm fractionation, 3 x 10^6 H520 and H82 cells were collected and resuspended in 300 µL of lysis buffer A (Tris-HCl pH 7.0 10 mM; NaCl 140 mM; $MgCl_2$ 1.5 mM; NP-40 0.5%; and deionized water), left on ice for 5 minutes and then centrifuged. The supernatant (*i.e.,* cytoplasm) was used for RNA extraction, while the pellet (*i.e.*, nucleus) was washed three times with 300 µL of lysis buffer A and then resuspended in 300 µL of lysis buffer B (Tris-HCl pH 7.0 10 mM; NaCl 140 mM; $MgCl_2$ 1.5 mM; NP-40 0.5%; Tween-20 1%; deoxycholic acid 0.5%; and deionized water). After centrifugation, the pellet was used for RNA extraction. All reactions were carried out on ice and centrifugations at 1,000 g for 3 minutes at 4°C. cDNA of the nuclear and cytoplasmic fractions was analyzed through qRT-PCR to evaluate the percentage of *NFYC-AS1* expression in the nucleus and cytoplasm. The nuclear lncRNA *MALAT1* and the cytoplasmic protein coding *GAPDH* were used as positive controls for either fraction.

Coding potential

The coding potential of *NFYC-AS1* was investigated through ORF Finder (4), and different alignment-free tools, including CPAT (5), CNIT (6), and LGC (7), to search for possible open reading frames (ORFs), using *NFYC-AS1* reconstructed annotation as input. Additionally, the translatome module of POSTAR (8) was used to assess the translational landscape based on Ribo-seq data of selected cell lines and tissues (Supplementary Figure S3C and Supplementary Tables S6-10).

RNA extraction, retrotranscription, and qRT-PCR

Total RNA was extracted with the miRNeasy Mini Kit (QIAGEN, Hilden, Germany) and DNAse I (QIAGEN, Hilden, Germany), according to manufacturer's instructions. RNA was reverse transcribed into cDNA using the M-MLV Reverse Transcriptase kit (GeneSpin Srl, Milan, Italy). When specifically stated, RNA was reverse transcribed into cDNA using either only random hexanucleotide primers or oligo-d$(T)_{16}$ primer (Invitrogen™ by Thermo Fisher Scientific Inc., Waltham, MA, USA) with the High-Capacity cDNA Reverse Transcription Kit with RNase Inhibitor (Applied Biosystems by Thermo Fisher Scientific Inc., Waltham, MA, USA).

Gene expression level was assessed through qRT-PCR using SsoAdvanced™ Universal SYBR Green Supermix (Bio-Rad, Hercules, CA, USA) and Bio-Rad CFX Connect™ RT-PCR instrument using the ribosomal protein S20 (*RPS20*) as endogenous gene. Gene expression level was calculated using the ΔΔCt method and expressed as relative quantity (rq). Rq values of each replicate experiment are reported in Original Data 1.

Primers were designed with Primer3Plus software (17) and are listed in Supplementary Table S1. To properly measure *NFYC-AS1* expression level and given the impossibility to design exon-spanning

primers due to *NFYC-AS1* monoexonic nature, we designed different primers covering the entire length of the transcript but not overlapping with *NFYC* (Supplementary Table S1).

Transcript decay

To study the transcript decay, 2 x 10^6 H520 and H82 cells were seeded in 60 mm and 35 mm dishes per time point, respectively. The day after, cells were treated with Actinomycin-D (Sigma-Aldrich, Saint Louis, MI, USA) at a final concentration of 10 µg/mL for 0, 1, 2, 4, and 8 hours. RNA extracted and retrotranscribed into cDNA of treated cells at different time points after recovery was analyzed through qRT-PCR to evaluate the decay of genes of interest. The normalization was done against the 0 h time point for each tested gene and c-*MYC* was used as a positive control, as it is a short-lived transcript.

Target accessibility analysis

The target accessibility analysis was based on the prediction of the structure of the RNA through Sfold (9). The estimated accessibility of each Gapmer-targeted region was calculated as the average of the accessibility percentage of each single base belonging to the *NFYC-AS1* portions targeted by the different Gapmers. The NCBI RefSeq *NFYC-AS1* annotation with the proximal TSS and the distal TSS was used as input for this analysis. Additionally, RNAfold web service (10) was used to obtain *NFYC-AS1* predicted secondary structure.

Cell proliferation assay

H520 cells, WT and ΔTSS H520 clones were seeded in 6-well plates at a density of 9 x 10^4 cells/well, whereas H82 cells were seeded 1.0 x 10^6 cells per T25 flask. The total/viable cell number was counted at 72, 96, and 120 hours after transfection/seeding with the TC20™ Automated Cell Counter (Bio-Rad, Hercules, CA, USA). Data were normalized against NEG/WT at 72h.

For the confluency experiment, H520 cells were plated in 60 mm petri at different density, 2.0 x 10^5, 4.0 x 10^5, 8.0 x 10^5, and 12.0 x 10^5, and collected after 4 days for RNA extraction and qRT-PCR measurement. C*CND1* RNA levels were used as marker of proliferation.

Cell cycle FACS analysis

Wild-type H520 cells, non-deleted and deleted clones were harvested, washed with PBS Dulbecco's Phosphate Buffered Saline (Euroclone S.p.A., Pero, Italy) and fixed with 70% ethanol. Cells were then washed twice with PBS and incubated in propidium iodide solution (PI 50 µg/ml, RNAse A 1 µg/ml, Tween 20 0.1%) for 30 min in the dark. DNA content was analyzed by BD Accuri™ C6 flow cytometer and BD CSampler Analysis Software (Becton Dickinson, Franklin Lakes, NJ, USA). At least 30,000 events were read, and cell cycle analysis was performed on ModFIT LT™ software according to the Modfit model (Becton Dickinson).

Survival analysis

We retrieved Progression Free Interval (PFI) time records of TCGA LUSC patients from the UCSC Xena web page (18) and overall survival (OS) of SCLC patients from GSE60052. We employed the Cutoff Finder tool (11) to find the optimal threshold for dichotomization of tumor samples based on the

NFYC-AS1 expression and survival data. Survival analysis was performed according to the Kaplan-Meier analysis and a two-sided log-rank test using the *survfit* function from the survival R package. The survival curve was plotted using *ggsurvplot* function from survminer R package.

TransCistor

Transcistor tool (14) was used to investigate the *in cis* and *in trans* modes of action of a *NFYC-AS1* based on enrichment of targets amongst proximal and distal genes. Input files are DEseq2 list of differentially regulated genes and unchanged genes in each Gapmers (GAP2-GAP4) versus the Negative Control Gapmer, and in ΔTSS clones versus WT clones.

Immunoblotting analysis

For total protein extraction, cells were lysed in RIPA buffer (10mM Tris-HCl pH 8.0, 1 mM EDTA, 0.5 mM EGTA, 0.1% SDS, 0.1% Deoxycholic acid, 1% Triton X- 100, with the addition of 0.1% PIC (Roche by Sigma-Aldrich, Saint Louis, MI, USA) and 0.1% PMSF (Sigma-Aldrich, Saint Louis, MI, USA) protease inhibitors) for 30 min on ice and then supernatant (*i.e.,* proteins) was boiled at 95 °C for 5 min. Proteins were loaded onto a 4-12% SDS-polyacrylamide gels (stacking gel: 125 mM Tris HCl pH= 6.8, 4% acrylamide:bis-acrylamide solution 37.5:1 (Euroclone S.p.A., Pero, Italy), 0.1% SDS, 0.1% APS, 0.01% Temed; separating gel: 375 mM Tris HCl pH= 8.8, 12% acrylamide:bis-acrylamide solution 37.5:1 (Euroclone S.p.A., Pero, Italy), 0.1% SDS, 0.1% APS, 0.01% Temed). Electrophoretic run was conducted at constant voltage in Running Buffer (25 mM Tris-Base, 190 mM Glycine, 0.1% SDS). Samples were transferred to a nitrocellulose membrane at constant current at 4°C for 2 hours in Transfer Buffer (25 mM Tris-Base, 190 mM Glycine, 20% methanol), using BioRad Mini-PROTEAN® Tetra Cell 4-Gel System. Nitrocellulose membranes were blocked for 1 hour shaking incubation in 5% skimmed milk solution in TBS-T (50 mM Tris-Base, 150 mM NaCl, 0.1% Tween 20) at room temperature. Membranes were incubated with the primary antibody overnight at 4°C in continuous shaking. Primary antibodies were prepared in 5% skimmed milk solution in TBS-T according to recommended dilution (Supplementary Table S13). Incubated membranes were washed three times in TBS-T for 10 minutes with shaking at room temperature and incubated in the same conditions with secondary antibody for 1 hour. Peroxidase-conjugated secondary antibodies (Sigma-Aldrich, Saint Louis, MI, USA) were prepared 1:10,000 in 5% skimmed milk solution in TBS-T. Membranes were washed three times in TBS-T for 10 minutes with shaking at room temperature and briefly incubated in peroxidase solution (1:1 of Peroxidase and Enhancer Solution purchased by GeneSpin Srl, Milan, Italy). Immunoreactive bands were visualized using Bio-Rad ChemiDoc MP, with ImageLab software. Vinculin was used as housekeeping protein for normalization and quantification of bands intensity using ImageJ software. Full blots are displayed in Original Data 2.

Analysis of RNA-seq data

Differential expression analysis for each Gapmer compared with NEG and for ΔTSS clones compared with WT clones was performed using the R package DESeq2. Gene set enrichment analysis was performed on genes ranked by DESeq2 stat values by GSEA Pre-ranked (12) using GSEA Hallmark collection. The common enriched pathways for all the three Gapmers were considered and the heatmaps and bubble plot of the significant normalized enrichment scores (NES) when the false

discovery rate (FDR) was lower than 0.10 for all the three Gapmers and clones were plotted using ComplexHeatmap and ggplot2 R packages, respectively. The fold-change (log_2FC) of common leading-edge genes of *UV response DN* and *G2/M checkpoint* gene sets (Supplementary Table S12) were plotted together with the relative tumor/normal ratio (expressed as log_2FC) in LUAD, LUSC (TCGA) and SCLC (GSE60052) cohorts. Moreover, GSEA C2 Reactome collection was tested on ranked gene lists of all Gapmers versus NEG and ΔTSS versus WT clones, and the relative bar plot of significant NES (FDR<0.05 in all the three Gapmers and clones) was plotted using the R software.

Ranked gene list for Gapmers versus NEG was tested on GSEA C2 collection (specifically FISCHER G1/S CELL CYCLE, FISCHER G2/M CELL CYCLE, WHITFIELD CELL CYCLE G1/S, and WHITFIELD CELL CYCLE G2/M gene sets) and on other custom gene sets, including lung cancer vulnerabilities as from *Sauta E et al*. (19), and genes synthetic lethal in *RB1*-mutated cells as from *Oser MG et al*. (20). Moreover, we defined lists of confident targets for activator E2F factors, repressor E2F factors, FOXM1, MYBL2, and NF-Y, all known to take part in the transcriptional control of the different cell cycle phases (21–24). We downloaded ChIP-seq data for these factors from ENCODE (FOXM1: GSE209315; MYBL2: GSE170799; NF-Y: GSE231181, GSE170375, GSE170694, GSE96244; E2F1/2/3: GSE231164, GSE169862, GSE169833, GSE169964; E2F4/5: GSE231060, GSE170651, GSE230897, GSE170241) (25), which were then aligned to NCBI RefSeq (GRCh38/hg38) annotation in order to get the list of genes having a peak in the promoter region. We also retrieved genomic positions of the consensus motif of these factors in the promoter region using *findMotif* tool downloaded from the UCSC Genome Browser Downloads page (26). Thus, confident targets were defined by the presence of ChIP-seq peak and the specific consensus motif in the promoter region, always defined as -1,000/+1,000 form the TSS. We used TTTSSCGC consensus motif for activator E2F1/2/3, TTTSSCGC and/or CHR (TTYRAA) consensus motif for repressor E2F4/5, CHR (TTYRAA) consensus motif for FOXM1 and MYBL2 (21,22), and CCAAT consensus motif for NF-Y (27). Target gene lists of these factors (reported in Supplementary Table S14) were used for GSEA analysis using ranked gene list for Gapmers versus NEG.

SUPPLEMENTARY REFERENCES

1. Lonsdale J, Thomas J, Salvatore M, Phillips R, Lo E, Shad S, et al. The Genotype-Tissue Expression (GTEx) project. Nat Genet. 2013 Jun;45(6):580–5.

2. Weinstein JN, Collisson EA, Mills GB, Shaw KRM, Ozenberger BA, Ellrott K, et al. The Cancer Genome Atlas Pan-Cancer analysis project. Nat Genet. 2013 Oct;45(10):1113–20.

3. Barretina J, Caponigro G, Stransky N, Venkatesan K, Margolin AA, Kim S, et al. The Cancer Cell Line Encyclopedia enables predictive modelling of anticancer drug sensitivity. Nature. 2012 Mar 29;483(7391):603–7.

4. Wheeler DL, Church DM, Federhen S, Lash AE, Madden TL, Pontius JU, et al. Database resources of the National Center for Biotechnology. Nucleic Acids Res. 2003 Jan 1;31(1):28–33.

5. Wang L, Park HJ, Dasari S, Wang S, Kocher JP, Li W. CPAT: Coding-Potential Assessment Tool using an alignment-free logistic regression model. Nucleic Acids Res. 2013 Apr 1;41(6):e74.

6. Guo JC, Fang SS, Wu Y, Zhang JH, Chen Y, Liu J, et al. CNIT: a fast and accurate web tool for identifying protein-coding and long non-coding transcripts based on intrinsic sequence composition. Nucleic Acids Res. 2019 Jul 2;47(W1):W516–22.

7. Wang G, Yin H, Li B, Yu C, Wang F, Xu X, et al. Characterization and identification of long non-coding RNAs based on feature relationship. Bioinformatics. 2019 Sep 1;35(17):2949–56.

8. Hu B, Yang YCT, Huang Y, Zhu Y, Lu ZJ. POSTAR: a platform for exploring post-transcriptional regulation coordinated by RNA-binding proteins. Nucleic Acids Res. 2017 Jan 4;45(D1):D104–14.

9. Ding Y, Chan CY, Lawrence CE. Sfold web server for statistical folding and rational design of nucleic acids. Nucleic Acids Res. 2004 Jul 1;32(suppl_2):W135–41.

10. Gruber AR, Lorenz R, Bernhart SH, Neuböck R, Hofacker IL. The Vienna RNA Websuite. Nucleic Acids Res. 2008 Jul 1;36(suppl_2):W70–4.

11. Budczies J, Klauschen F, Sinn BV, Győrffy B, Schmitt WD, Darb-Esfahani S, et al. Cutoff Finder: A Comprehensive and Straightforward Web Application Enabling Rapid Biomarker Cutoff Optimization. PLOS ONE. 2012 Dec 14;7(12):e51862.

12. Subramanian A, Tamayo P, Mootha VK, Mukherjee S, Ebert BL, Gillette MA, et al. Gene set enrichment analysis: A knowledge-based approach for interpreting genome-wide expression profiles. Proc Natl Acad Sci. 2005 Oct 25;102(43):15545–50.

13. Hao Q, Zong X, Sun Q, Lin YC, Song YJ, Hashemikhabir S, et al. The S-phase-induced lncRNA SUNO1 promotes cell proliferation by controlling YAP1/Hippo signaling pathway. Davis RJ, Struhl K, editors. eLife. 2020 Oct 27;9:e55102.

14. Dhaka B, Zimmerli M, Hanhart D, Moser MB, Guillen-Ramirez H, Mishra S, et al. Functional identification of cis-regulatory long noncoding RNAs at controlled false discovery rates. Nucleic Acids Res. 2024 Feb 13:gkae075.

15. Girard L, Rodriguez-Canales J, Behrens C, Thompson DM, Botros IW, Tang H, et al. An Expression Signature as an Aid to the Histologic Classification of Non-Small Cell Lung Cancer. Clin Cancer Res. 2016 Oct 1;22(19):4880–9.

16. Ireland AS, Micinski AM, Kastner DW, Guo B, Wait SJ, Spainhower KB, et al. MYC Drives Temporal Evolution of Small Cell Lung Cancer Subtypes by Reprogramming Neuroendocrine Fate. Cancer Cell. 2020 Jul 13;38(1):60-78.e12.

17. Untergasser A, Nijveen H, Rao X, Bisseling T, Geurts R, Leunissen JAM. Primer3Plus, an enhanced web interface to Primer3. Nucleic Acids Res. 2007 Jul;35(Web Server issue):W71-74.

18. Goldman MJ, Craft B, Hastie M, Repečka K, McDade F, Kamath A, et al. Visualizing and interpreting cancer genomics data via the Xena platform. Nat Biotechnol. 2020 Jun;38(6):675–8.

19. Sauta E, Reggiani F, Torricelli F, Zanetti E, Tagliavini E, Santandrea G, et al. CSNK1A1, KDM2A, and LTB4R2 Are New Druggable Vulnerabilities in Lung Cancer. Cancers. 2021 Jul 12;13(14):3477.

20. Oser MG, Fonseca R, Chakraborty AA, Brough R, Spektor A, Jennings RB, et al. Cells Lacking the RB1 Tumor Suppressor Gene Are Hyperdependent on Aurora B Kinase for Survival. Cancer Discov. 2019 Feb 8;9(2):230–47.

21. Fischer M, Schade AE, Branigan TB, Müller GA, DeCaprio JA. Coordinating gene expression during the cell cycle. Trends Biochem Sci. 2022 Dec 1;47(12):1009–22.

22. Fischer M, Grossmann P, Padi M, DeCaprio JA. Integration of TP53, DREAM, MMB-FOXM1 and RB-E2F target gene analyses identifies cell cycle gene regulatory networks. Nucleic Acids Res. 2016 Jul 27;44(13):6070–86.

23. Sadasivam S, DeCaprio JA. The DREAM complex: Master coordinator of cell cycle dependent gene expression. Nat Rev Cancer. 2013 Aug;13(8):585–95.

24. Dolfini D, Zambelli F, Pedrazzoli M, Mantovani R, Pavesi G. A high definition look at the NF-Y regulome reveals genome-wide associations with selected transcription factors. Nucleic Acids Res. 2016 Jun 2;44(10):4684–702.

25. Dunham I, Kundaje A, Aldred SF, Collins PJ, Davis CA, Doyle F, et al. An integrated encyclopedia of DNA elements in the human genome. Nature. 2012 Sep;489(7414):57–74.

26. Navarro Gonzalez J, Zweig AS, Speir ML, Schmelter D, Rosenbloom KR, Raney BJ, et al. The UCSC Genome Browser database: 2021 update. Nucleic Acids Res. 2021 Jan 8;49(D1):D1046–57.

27. Dolfini D, Gatta R, Mantovani R. NF-Y and the transcriptional activation of CCAAT promoters. Crit Rev Biochem Mol Biol. 2012 Feb 1;47(1):29–49.
